# Schizophrenia-Associated *MIR204* Regulates Noncoding RNAs and Affects Neurotransmitter and Ion Channel Gene Sets

**DOI:** 10.1371/journal.pone.0144428

**Published:** 2015-12-29

**Authors:** Sophia Cammaerts, Mojca Strazisar, Bart Smets, Sarah Weckhuysen, Annelie Nordin, Peter De Jonghe, Rolf Adolfsson, Peter De Rijk, Jurgen Del Favero

**Affiliations:** 1 University of Antwerp, Antwerp, Belgium; 2 Applied Molecular Genomics Unit, Department of Molecular Genetics, VIB, Antwerp, Belgium; 3 Centralized Service Facility, Department of Molecular Genetics, VIB, Antwerp, Belgium; 4 Neurogenetics Group, Department of Molecular Genetics, VIB, Antwerp, Belgium; 5 Division of Psychiatry, Department of Clinical Sciences, Umeå University, Umeå, Sweden; 6 Antwerp University Hospital, Antwerp, Belgium; 7 Multiplicom N.V., Niel, Belgium; National University of Singapore, SINGAPORE

## Abstract

As regulators of gene expression, microRNAs (miRNAs) are likely to play an important role in the development of disease. In this study we present a large-scale strategy to identify miRNAs with a role in the regulation of neuronal processes. Thereby we found variant rs7861254 located near the *MIR204* gene to be significantly associated with schizophrenia. This variant resulted in reduced expression of miR-204 in neuronal-like SH-SY5Y cells. Analysis of the consequences of the altered miR-204 expression on the transcriptome of these cells uncovered a new mode of action for miR-204, being the regulation of noncoding RNAs (ncRNAs), including several miRNAs, such as *MIR296*. Furthermore, pathway analysis showed downstream effects of miR-204 on neurotransmitter and ion channel related gene sets, potentially mediated by miRNAs regulated through miR-204.

## Introduction

miRNAs are short regulatory noncoding RNA molecules. Their maturation process consists of two consecutive cleavage steps. The first step takes place in the nucleus, whereby the Microprocessor complex, consisting of ribonuclease Drosha and its cofactor DGCR8, frees the hairpin structured precursor miRNA (pre-miRNA) from the primary miRNA transcript (pri-miRNA) [1,2]. After export to the cytosol by Exportin-5 [3], the ribonuclease Dicer cuts the terminal loop off the pre-miRNA [4,5]. Subsequently, the mature miRNA duplex, containing the 5p and 3p mature miRNA strands, is loaded onto the RNA-induced silencing complex (RISC). One of the strands is retained and will guide Argonaute, the active compound of RISC, to its targets via incomplete sequence complementarity [6,7]. Posttranscriptional regulation of messenger RNAs (mRNA) in the cytosol by miRNAs is well studied [8]. Next to this canonical cytoplasmic role of miRNAs, mature miRNA-Argonaute complexes can be shuttled back into the nucleus by Importin-8 [9]. Many mature miRNAs have been shown to be present in the nuclear as well as the cytoplasmic fraction of cells [10], indicating functionality in the nucleus. Recently, it was also shown that though mRNAs are the main targets of miRNA regulation, as much as 30% of miRNA interactions take place with other classes of RNAs [11].

Identification of miRNAs involved in disease pathomechanisms is often performed at the RNA level, by comparing miRNA expression profiles in affected tissues between individuals with different phenotypes or in affected versus nonaffected tissue of patients [12,13]. The miRNAs that are differentially expressed between the studied states then indicate which miRNAs may be functionally relevant for the disease. However, from such analysis it is not possible to derive whether the miRNA is a primary factor in the process or a downstream effect [14]. In addition, for diseases where the affected patient tissue is not readily available, the profiling approach is not applicable. Another starting point is a genetic approach, where one searches for causal or risk factors associated with disease in (miRNA) genes. The miRNA genes with associated or causal variants can then be further investigated to assess the functional effects of these variants [15,16].

Here we describe a high throughput genetic approach for the identification of miRNAs regulating tissue-specific functions. This strategy is based on a large-scale targeted miRNA gene variant screening and association analysis with disease phenotypes related to the tissue of interest. Variants enriched in these phenotypes are then functionally investigated to study the wild type function of these miRNA genes. We applied this strategy to identify miRNAs with a function in neuronal pathways by variant discovery in brain expressed miRNA genes in patients with neurological disorders: schizophrenia and idiopathic generalized epilepsy (IGE). Two selected miRNA gene variants were functionally validated in the neuronal-like cell line SH-SY5Y.

## Results

### Large-scale variant screening

To study the function of miRNA genes in neurological diseases and to investigate which miRNA genes regulate neuronal processes, we created a Multiplex Amplification of Specific Targets for Resequencing (MASTR) assay [17,18] for the amplification of 289 brain expressed miRNA genes. This assay was subsequently used in combination with massively parallel sequencing for variant discovery within the selected miRNA genes in patients with schizophrenia or IGE and in Swedish and Belgian and Dutch control individuals. After mapping, variant calling and variant filtering, an association analysis was performed to assess which variants are over- or underrepresented in the disease phenotypes and thus may have a neuronal function (S1 File). After filtering, a total of 265 variants were identified in the schizophrenia patients and the Swedish control group and 315 variants were identified in the IGE patients and the Belgian/Dutch control group (Tables A and B in S1 File). In both patient groups, individual patients had on average 45 different variants in or near miRNA genes (Figure A in S1 File). In the schizophrenia group, ten variants had a difference in variant frequency between the patients and controls (unadjusted p < 0.05). One of these variants, rs7861254, was significantly associated with schizophrenia after Bonferroni correction. In the IGE group, eight variants had a difference in frequency in the patients compared to the controls (Tables C and D in S1 File). All variants with (unadjusted) p-value < 0.05 were considered for assessment of variant location relative to the functional miRNA regions and structural impact prediction using the miRVaS software [19].

**Fig 1:**
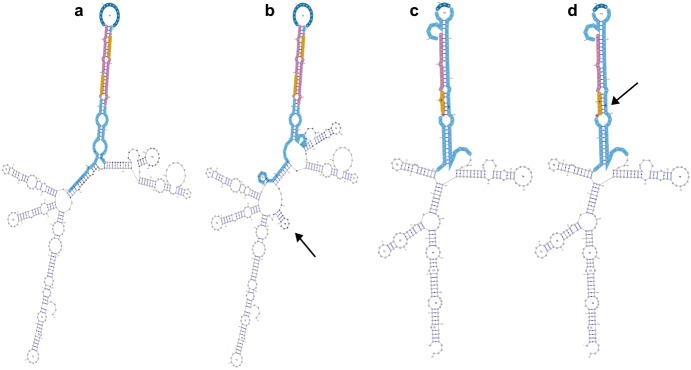
MFE secondary structure predictions of hsa-mir-204 and hsa-mir-618 as generated by miRVaS. (a-b) MFE structure for hsa-mir-204 with variant rs7861254, (a) wild type miRNA, (b) variant miRNA. As the variant is located 107 nt outside the hairpin, flanking regions of 150 nt were included for the predictions. Centroid and MEA predictions with this flank size also showed large structural changes (but different changes), while predictions with flanks of 200 nt resulted in minor changes far away from the hairpin. (c-d) MFE structure of hsa-mir-618 for variant rs2682818, (c) wild type miRNA, (d) variant miRNA. The variant is predicted to induce a shift of the first base of the miR-618 sequence into an internal loop. Flanks of 100 nt were used for this prediction. Predictions were also run for the hairpin with flank sizes of 50 nt, 150 nt and 200 nt and centroid and MEA structures: all predicted the same change within the hairpin. Color scheme: magenta: mature miRNA, orange: seed region, dark blue: terminal loop, cyan: hairpin. The variant is colored in red and indicated by an arrow. Structural changes induced by the variant are colored in black.

Based on the association analysis and impact prediction, two variants were selected for further functional studies. The first variant, rs7861254 C>T, showed a significant lower variant frequency in schizophrenia patients compared to Swedish control individuals (alternative allele frequency patients / controls: 16.39% / 25.34%, unadjusted p = 1.51x10^-4^; Bonferroni adjusted p = 0.04). Although the variant is located 107 nt outside the *MIR204* hairpin, it is predicted to induce structural changes in the hairpin end and flanking regions, which can have an effect on its processing (Fig 1A and 1B). The second variant, rs2682818 A>C, is more frequently present in IGE patients than in Belgian/Dutch controls (alternative allele frequency patients / controls: 91.67% / 85.75%, unadjusted p = 9.63x10^-3^, Bonferroni adjusted p = 3.03) and is located in *MIR618*, in the pre-miR-618 hairpin arm opposite of the mature miR-618 sequence. Based on the secondary structure prediction, we hypothesized that the variant allele may affect the distribution pattern of canonical and alternative mature miR-618 sequences (isomiRs), as it was predicted to induce a structural change at the site of Drosha cleavage (Fig 1C and 1D). This structural change was very consistent between different types of structure predictions (minimal free energy (MFE), centroid, maximal expected accuracy (MEA)) and with varying flank sizes.

### Functional characterization of *MIR204*

#### Variant rs7861254 results in altered processing of miR-204

**Fig 2:**
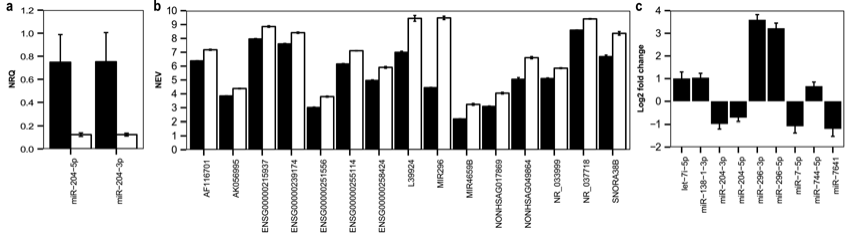
QPCR (a), microarray (b) and small RNA sequencing results (c) for *MIR204* cells. (a) Average normalized relative quantity (NRQ) for miR-204-5p and miR-204-3p in MIR204WT (CC genotype, black bars, clones 1–3) and MIR204SNP cells (TT genotype, white bars, clones 1–3) normalized for transduction efficiency. (b) Average normalized expression values (NEV) for significantly differently expressed genes between MIR204WT (CC genotype, black bars, clones 4–5) and MIR204SNP (TT genotype, white bars, clones 4–6) cells. (c) Log2 fold changes of miRNAs significantly differently expressed in MIR204SNP cells (clones 4–6) compared to MIR204WT cells (clones 4–5). Error bars in panels (a-b) represent standard deviation of biological replicates, error bars in (c) represent standard error as calculated by DESeq2.

To explore the functional effect of rs7861254 genotypes and to investigate *MIR204* function, we established stable cells overexpressing the wild type or mutant *MIR204* gene. As our aim was to study this gene in the context of neurological disease, we used the neuronal-like cell line SH-SY5Y for these experiments. We assessed the expression of both mature miRNAs (miR-204-5p and miR-204-3p) in replicates of wild type (MIR204WT) and mutant (MIR204SNP) cell lines by RT-qPCR and corrected expression for transduction efficiency. In a first set of triplicates of wild type and mutant cells, a lower expression of both mature miRNA sequences was measured in the mutant cells (miR-204-5p: fold change MIR204SNP versus MIR204WT = 0.166, p = 0.046; miR-204-3p: fold change MIR204SNP versus MIR204WT = 0.165, p = 0.050; Fig 2A, Figure B(a) in S1 File), confirming the predicted effect of the variant on the biogenesis of miR-204. A comparable trend was found in a set of three additional clones (Figures B(b) and C in S1 File, p > 0.05).

#### Transcriptome analysis

We next analyzed the effect of the variant-induced lowered expression of miR-204 on the transcriptome of the stable SH-SY5Y cells using microarray analysis to deduce the wild type function of *MIR204*. Significance Analysis of Microarray (SAM) analysis indicated that 15 genes are significantly differentially expressed in the mutant cells versus the wild type cells (Fig 2B, Table 1, Figure D in S1 File). Rather unexpectedly, these genes all code for ncRNAs. The gene with highest fold change is *MIR296*, which is upregulated 33-fold in the MIR204SNP cells.

**Table 1:** Genes significantly differentially expressed between MIR204WT and MIR204SNP cells, as identified by SAM.

Gene	Transcript	Type	dExp	d	FC
AK056995	n341034	mRNAlike lncRNA	0.316	10.674	1.45
NR_033999	n409616	lncRNA	-0.208	10.766	1.68
ENSG00000251556	ENST00000514002	antisense	-0.575	11.229	1.72
AF116701	n341807	mRNAlike lncRNA	-0.012	11.972	1.74
ENSG00000239174°	ENST00000459378°	miRNA	0.757	10.588	1.75
NR_037718	n410551	lncRNA	0.335	15.018	1.76
ENSG00000215937°	ENST00000401118°	miRNA	0.988	11.016	1.86
ENSG00000258424	ENST00000555853	processed transcript*	0.570	10.485	1.93
ENSG00000255114	ENST00000531886	antisense	0.261	16.937	1.94
NONHSAG017869^●^	n346045	lincRNA	0.701	11.235	1.95
MIR4659B	NR_039807	miRNA	-0.166	11.804	2.07
NONHSAG049864^●^	n379344	lncRNA**	-0.114	12.256	2.95
SNORA38B	NR_003706	small nucleolar RNA	0.824	11.157	3.21
L39924	n342713	mRNAlike lncRNA	0.913	12.294	5.50
MIR296	NR_029844	miRNA	1.251	39.205	32.97

Gene (or locus), transcript and transcript type information is derived from the HTA2.0 annotation file (HTA-2_0.na34.hg19.transcript), except for those indicated with:

^●^ NONCODE v4 gene symbol used.

° Retired transcript or gene

* Ensembl release 79: antisense

** NONCODE v4: antisense.

dExp: expected score, d: observed score as calculated by SAM. FC: fold change SNP versus WT as calculated by SAM.

To investigate the effect of miR-204 on whole pathways as opposed to separate genes, we also performed gene set enrichment analysis (GSEA). GSEA was performed with Gene Ontology gene sets for molecular function, comparing MIR204WT to MIR204SNP cells (Tables 2 and 3). 30 gene sets were significantly enriched in MIR204WT cells. The gene set with the strongest normalized enrichment score is neurotransmitter binding. In addition, several ion channel activity related gene sets are highly enriched. In MIR204SNP cells, 17 gene sets were significantly enriched. These sets were related to RNA and DNA binding and ligase activities, amongst other functions.

**Table 2:** Gene sets enriched in MIR204WT cells with FDR < 0.01 and nominal p-value < 0.001.

Gene set	Size	NES	Nominal p-value	FDR q-value
Neurotransmitter binding	52	2.316	0.000	0.001
Substrate specific channel activity	150	2.247	0.000	0.000
Neurotransmitter receptor activity	49	2.224	0.000	0.000
Ion channel activity	144	2.206	0.000	0.000
Potassium channel activity	47	2.155	0.000	0.000
Gated channel activity	119	2.127	0.000	0.001
Transmembrane receptor activity	404	2.113	0.000	0.001
Peptide receptor activity	53	2.101	0.000	0.000
G protein coupled receptor activity	189	2.099	0.000	0.000
Cation channel activity	114	2.099	0.000	0.000
Chemokine receptor binding	39	2.046	0.000	0.001
Neuropeptide binding	23	2.027	0.000	0.001
G protein coupled receptor binding	48	2.017	0.000	0.001
Voltage gated potassium channel activity	34	2.012	0.000	0.001
Metal ion transmembrane transporter activity	142	2.000	0.000	0.001
Chemokine activity	38	1.999	0.000	0.001
Neuropeptide receptor activity	22	1.981	0.000	0.001
Monooxygenase activity	30	1.938	0.000	0.002
Rhodopsin like receptor activity	134	1.915	0.000	0.003
Ligand gated channel activity	39	1.910	0.000	0.003
Serine type endopeptidase activity	37	1.896	0.000	0.003
Carbohydrate binding	69	1.884	0.000	0.003
Substrate specific transmembrane transporter activity	330	1.868	0.000	0.005
Ion transmembrane transporter activity	267	1.868	0.000	0.004
Serine type peptidase activity	41	1.863	0.000	0.004
Transmembrane transporter activity	360	1.862	0.000	0.004
Substrate specific transporter activity	374	1.836	0.000	0.005
Serine hydrolase activity	42	1.831	0.000	0.005
Cation transmembrane transporter activity	204	1.814	0.000	0.006
Voltage gated cation channel activity	64	1.808	0.000	0.006

NES: normalized enrichment score, FDR: false discovery rate.

**Table 3:** Gene sets enriched in MIR204SNP cells with FDR < 0.01 and nominal p-value < 0.001.

Gene set	Size	NES	Nominal p-value	FDR q-value
Structural constituent of ribosome	59	-2.554	0.000	0.000
RNA binding	221	-2.430	0.000	0.001
Ligase activity forming carbon nitrogen bonds	66	-2.400	0.000	0.001
Acid amino acid ligase activity	55	-2.369	0.000	0.000
Single stranded DNA binding	29	-2.361	0.000	0.000
Ligase activity	95	-2.329	0.000	0.000
Ubiquitin protein ligase activity	48	-2.292	0.000	0.000
Small conjugating protein ligase activity	49	-2.281	0.000	0.000
Small protein conjugating enzyme activity	51	-2.280	0.000	0.000
Structure specific DNA binding	49	-2.147	0.000	0.003
RNA splicing factor activity transesterification mechanism	18	-2.076	0.000	0.006
Nucleotidyltransferase activity	44	-2.062	0.000	0.007
Helicase activity	48	-2.046	0.000	0.007
Translation factor activity nucleic acid binding	32	-2.041	0.000	0.006
Translation regulator activity	34	-2.000	0.000	0.008
Protein serine threonine kinase activity	195	-1.993	0.000	0.008
Tubulin binding	44	-1.986	0.000	0.008

Since several ncRNAs are significantly upregulated in MIR204SNP cells, it is plausible to assume a more direct relationship between the RNA, DNA binding and ligase gene sets and miR-204 function and an indirect relationship with the neurotransmitter and ion channel gene sets, possibly via miR-296 or any of the other ncRNAs that are differentially expressed in the mutant cells.

#### Genome-wide mature miRNA expression analysis

To evaluate the effect of miR-204 on mature miRNA expression, the cells were also subjected to small RNA sequencing. Differential miRNA expression analysis using DESeq2 [20] indicated seven mature miRNAs that are significantly differently expressed between MIR204WT and MIR204SNP cells, in addition to miR-204-5p and miR-204-3p (Benjamini-Hochberg corrected p-value < 0.1; Fig 2C). Consistent with the microarray results where *MIR296* is strongly upregulated, miR-296-5p and miR-296-3p are both significantly upregulated (9- and 12-fold) in MIR204SNP cells. Furthermore, consistent with the presence of other ncRNAs being upregulated according to the array results, five other mature miRNAs are deregulated (three upregulated, two downregulated), indicating that this might be a common function of *MIR204*.

#### Target prediction

To assess whether the differentially expressed transcripts contain binding sites for miR-204-5p or miR-204-3p regulation, we predicted the presence of seed binding sites using the RNAhybrid software [21]. Because the primary transcript length of miRNAs is not known in most cases, we included a flanking region of 200 nt surrounding the miRNA hairpin. Of the 18 transcripts tested, nine contain at least one seed match, including pri-miR-296 (S5 Table). Of these, pri-let-7i has the highest number of potential binding sites (after correction for transcript length), two for miR-204-5p and one for miR-204-3p. Though the presence of a seed match can further strengthen the finding that they are regulated by miR-204, the absence of a seed match not necessarily weakens it since it has been shown recently that miRNA-target interactions without a seed match are quite common [11].

### Functional characterization of *MIR618*

To investigate the effect of the variant rs2682818 and the function of *MIR618*, stable SH-SY5Y cells overexpressing wild type or mutant *MIR618* were established and subjected to the same analyses as *MIR204* cells. The expression level of miR-618 in the MIR618WT cells was too low to determine normalized relative quantities (NRQ) of miR-618 between MIR618WT and SNP cells by RT-qPCR. We reasoned this could either be due to very low or no expression of miR-618 in MIR618WT cells and/or due to the expression of an isomiR that cannot be detected by the assay used for RT-qPCR.

SAM analysis in *MIR618* cells failed to identify genes that were significantly differentially expressed in the mutant cells compared to the wild type cells (Figure E in S1 File). GSEA showed three gene sets to be significantly enriched in MIR618SNP cells (Table 4): helicase activity, taste receptor activity and ATPase activity. No pathways were enriched in MIR618WT cells with FDR < 0.05.

**Table 4:** Gene sets enriched in MIR618SNP cells with FDR < 0.01 and nominal p-value < 0.001.

Gene set	Size	NES	Nominal p-value	FDR q-value
Helicase activity	48	-2.222	0.000	0.003
Taste receptor activity	15	-2.147	0.000	0.006
ATPase activity	108	-2.087	0.000	0.006

Differential miRNA gene expression analysis on the small RNA sequencing data indicated that miR-618 was significantly differentially expressed with a 10-fold change. Because the secondary structure predictions indicated a potentially altered isomiR distribution, we investigated whether this was indeed the case. For this analysis, we used the raw read counts of all reads mapping to the miR-618 locus and grouped them in two classes: reads with the same 5' start as the canonical miRNA sequence as deposited in miRBase [22] (termed “5’ canonical isomiRs”) and reads with a different 5' start position (termed “5’ change isomiRs”), as these might have a different functionality. Per class the reads were summed and normalized to the total read count on the miR-618 locus within the sample. The MIR618SNP cells, that have a higher expression of miR-618, show a significantly reduced frequency of 5' change isomiRs compared to MIR618WT cells (p = 0.040; Figure F in S1 File). Given that the miR-618 expression in MIR618WT cells is very low (20–75 reads in total), the increased relative presence of 5' isomiRs may mean that these reads are degradation products rather than functional isomiRs. For comparison, we did the same isomiR analysis for miR-204-5p and miR-204-3p in *MIR204* cells (Figure F(b and c) in S1 File. The frequency of 5' change isomiRs of miR-204 was not significantly different between *MIR204* genotypes (miR-204-5p: p = 0.146, miR-204-3p: p = 0.573), indicating a *MIR618* genotype-dependent specific event.

Given that miR-618 expression is upregulated in the MIR618SNP cells, the enriched gene sets are likely indirect targets of miR-618 since their expression is positively correlated with miR-618 expression. As no genes were significantly differently expressed after a 10-fold upregulation of miR-618, gene sets were only enriched in the SNP cells and the expression of miR-618 in MIR618WT cells was very low, this may indicate that miR-618 has no direct targets in this specific cell type, and that ectopic overexpression results in dysregulation of downstream pathways.

## Discussion

We present a novel large-scale approach for the identification of miRNA genes involved in neuronal functions. The identification of relevant miRNA genes is based on an association study of variants in brain expressed miRNA genes in patient groups with neurological phenotypes and was performed here on schizophrenia and IGE. The genetic association of a miRNA variant with one of the phenotypes is employed as an indication for a miRNA to have a potential role in the regulation of neuronal pathways. This allowed us at the same time to identify genes with a potential function in neuronal processes and to identify variants that may be significantly associated with the studied phenotypes, such as the variant rs7861254 that is associated with schizophrenia. Functional validation of the selected miRNA genes was also performed on a large scale, implementing both genome-wide mRNA and miRNA profiling, to assess all possible targets of these genes and to assess whether the variants had any effect on the isomiR distribution pattern of the miRNA. This approach led to interesting and different findings for *MIR204* and for *MIR618*. The strategy can be adapted to identify miRNAs involved in a wide range of other organ- or tissue-specific functions, provided phenotypes are known, where the tissue or organ of interest is affected, and are available for genetic variant screening.

*MIR204* is located within an intron of *TRPM3*, which encodes for a transient receptor potential cation channel that plays a role in detecting noxious heat and chemical stimuli in sensory neurons [23]. Several cancer-related studies have investigated the function of *MIR204*. miR-204-5p was shown to have a tumor-suppressor function through the negative regulation of cell migration and invasion [24–27]. Its expression is reduced in different cancers, such as endometrial cancer, glioma, retinoblastoma and colorectal cancer [24–27]. miR-204-5p was demonstrated to regulate expression of *TRPM3* both directly and indirectly in renal cell carcinoma cell lines, as part of a network involved in regulation of autophagy [28]. miR-204 also plays an important role in the eye. A causal mutation in the miR-204-5p seed sequence was recently found in a family with inherited retinal dystrophy and coloboma [29]. In vivo studies in medaka fish implicated miR-204 in photoreceptor differentiation and function [29]. Previously, miR-204 had already been shown to play a crucial role in development of the lens and retina in medaka fish [30]. Transcriptome analysis in whole embryos also showed nervous system development, neurogenesis and axon guidance pathways to be differentially regulated when miR-204 expression was manipulated [31]. Its role in axonal outgrowth of retinal ganglion cells was further validated in vivo. A general mode of action of miR-204-5p appears to be the regulation of transcription factors. For example, miR-204-5p has been shown to regulate a network of transcription factors, MEIS2, FOXC1, PAX6, involved in eye development [30,32] and it targets the insulin transcription factor MAFA in pancreatic beta-cells [33]. In addition, one third of known miR-204-5p targets (9/27) listed in miRTarBase 4.5 [34] (only taking into account those interactions directly validated using reporter assays) are members of different transcription factor families, confirming this to be an important function of miR-204-5p.

The variant rs7861254 has not yet been functionally investigated for its effect on miR-204 expression. The alternative allele is significantly less frequent in schizophrenia patients than in the control individuals. By functionally validating the effects of rs7861254, we show that miR-204 regulates other miRNAs and ncRNAs. It exerts its largest effect on miR-296: both the pri/pre-miR-296 (as measured on the array) as miR-296-5p and -3p (small RNA sequencing data) are significantly upregulated in the MIR204SNP cells that have reduced miR-204 expression. Functional validation of miR-618 on the other hand did not identify any significantly differentially expressed target genes, when applying the same approaches. To the best of our knowledge, a direct regulatory role for miR-204 of other miRNAs has not been described yet, though it is consistent with the known function of miR-204 to target transcription factors as it characterizes miR-204 as a regulator of different classes of gene expression regulators in the cell.

Next to mediating posttranscriptional regulation of mRNAs, miRNAs can target promoters of protein-coding target genes resulting in transcriptional activation or repression [35]. It is therefore likely that they can also mediate the same type of transcriptional regulation of miRNA genes [36], either by targeting transcription factors that regulate the expression of miRNAs, or by targeting miRNA genes directly. In addition, both maturation and stability of miRNAs have been shown to be regulated by other miRNAs: mouse specific miR-709 regulates miR-15a-16-1 biogenesis by targeting the primary transcript and preventing its processing to pre-miRNA [37], while base pairing between mature miR-107 and let-7 reduces their stability bidirectionally [38]. That these miRNA-miRNA interactions are not isolated events was shown by a large-scale analysis of direct miRNA-target interactions with the CLASH technology in HEK293 cells where 3% of all interactions occurred between miRNAs [11].

We did not find any transcription factors or other protein-coding genes to be significantly deregulated upon miR-204 expression manipulation and did find potential miR-204 binding sites in several miRNAs, pointing to a more direct role of miR-204 in regulation of other ncRNAs. Nevertheless, the possibility of intermediary targets, for instance those targets with altered protein expression but unaltered mRNA expression, cannot be ruled out completely. The upregulation of *MIR296* expression at different levels indicates that the regulation occurs upstream of the pre-miRNA to mature miRNA processing and may take place either at pri-miRNA processing or at the level of transcription. For the other mature miRNAs that have different expression in MIR204SNP cells, the pri/pre expression is not significantly affected. Both a biological and a technical reason may exist. It could be that there are indeed no significant alterations at the pri/pre-miRNA level, pointing toward regulation at a different step compared to *MIR296*, e.g. Dicer processing or miRNA destabilization. On the other hand, it could be that the changes at the pri/pre level are not large enough to be picked up by the technology used. Further experiments are required to determine the direct or indirect nature of these interactions and at which step they take place.

*MIR618* is located within the first intron of the gene *LIN7A*. LIN7 proteins are present in pre- and post-synaptic protein complexes and are involved in regulating neurotransmitter release and transport of NMDA receptor vesicles [39–42]. The function of *MIR618* has not been explored in detail yet. Overexpression of miR-618 in an anaplastic thyroid cancer cell line resulted in inhibition of proliferation, invasion and migration of these cells [43]. RIP-Chip analysis of HeLa cells transfected with a miR-618 mimic and subsequent network analysis indicated a potential role for *MIR618* in lymphomagenesis [44]. In the same study, the A allele (wild type allele) of rs2682818 was found to be associated with an increased risk for follicular lymphoma. In vitro validation in HeLa cells showed that the AA genotype results in decreased miR-618 expression compared to the alternative CC genotype.

In this study, we found that the variant rs2682818 has a higher frequency in patients with IGE compared to control individuals (though not statistically significant after correction for multiple testing). Validation in SH-SY5Y cells indicated that the homozygous variant genotype (CC) results in a significantly higher expression of miR-618 in SH-SY5Y cells compared to the AA genotype. In addition, the variant cells have a significantly lower frequency of 5’ change isomiRs than MIR618WT cells, which confirmed our structure prediction-based hypothesis that the variant may induce altered pre-miR-618 processing resulting in a changed isomiR distribution pattern. Nevertheless, the large expression change induced by the variant did not lead to expression changes of protein-coding or of other microRNA genes. The absence of deregulated miRNA genes shows that the miRNA-regulation mode of action is a finding specific to miR-204. On the other hand, the presence of many 5’ changed isomiRs and the lack of enriched gene sets with negative correlated expression despite a large change in expression of miR-618 between MIR618WT and SNP cells may indicate that miR-618 has no endogenous role in the studied cell type and that high ectopic expression of this miRNA results in side effects.

With this study we demonstrated the different effects of two selected genetic variants on miRNA functionality. The variant rs2682818 in *MIR618* resulted in increased miR-618 expression and in a different distribution of miR-618 isomiRs. The variant rs7861254 near *MIR204* is significantly associated with schizophrenia, identifying a new instance of a miRNA involved in schizophrenia etiology. By studying this variant, that reduced miR-204 expression, a new function of *MIR204* became apparent: the regulation of other ncRNAs. Though the mechanism of miRNAs regulating expression of other miRNAs has been described before, it remains as yet an underexplored functionality of miRNAs. The discovery of a new schizophrenia related miRNA gene regulating neurotransmitter and ion channel gene sets and with a novel mode of action shows the utility of our large scale miRNA gene sequencing and functional analysis approach and indicates that it will be useful for the study of miRNAs in other tissues as well.

## Methods

### Ethics statement

The study was approved by the Medical Ethical Committees of the universities of Umeå and Antwerp. Each individual signed an informed consent form for participation in the study.

### Samples

DNA of patients with schizophrenia, of patients with IGE and of control individuals was collected. The schizophrenia patients included in this study were recruited in Sweden (n = 186). Control individuals for this patient group were also recruited in Sweden (n = 1043), as part of the Betula study [45,46]. Patients with IGE were recruited in Belgium (n = 162). Control individuals for this patient group were recruited in Belgium and the Netherlands (n = 273).

### Variant screening

A MASTR assay that amplifies 289 brain expressed miRNA genes was created (assay available upon request). This assay was subsequently used to screen the genomic DNA of patients and control individuals. Control DNA samples were pooled per seven samples prior to amplification, patient samples were amplified individually. Libraries were sequenced on MiSeq (Illumina, CA, USA) using v3 chemistry. After performing filtering steps in GenomeComb [47] (S1 File), remaining variants were tested for association with the schizophrenic or epileptic phenotype using a two-sided Fisher’s exact test. All variants with p < 0.05 were subsequently analyzed using the miRVaS software [19] to assess their location in the miRNA (seed, mature, terminal loop, hairpin, flanks) and their predicted structural impact. Bonferroni adjusted p-values were calculated by multiplying the Fisher’s exact p-value with the number of tested variants per phenotype. Additional information can be found in S1 File.

### Generation of stable cell lines

Variant and wild type miRNA genes were cloned by amplifying the miRNA gene and flanking sequences of at least 130 bp on either side from genomic DNA of patients heterozygous for the variant using sequence specific primers with attachment sites for Gateway cloning. Bead-purified PCR products with homozygous wild type or variant genotype were inserted into the pLenti6/EGFP-V5 destination vector via Gateway cloning as described by Almeida-Souza et al. [48]. Generation and culture of stable SH-SY5Y cells was performed as described previously [49]. Cells were cultured in medium supplemented with 3 μg/ml Blasticidin S (InvivoGen, CA, USA) to select for stable cells. The SH-SY5Y cell line was purchased from Sigma-Aldrich (MO, USA, catalog number: 94030304-1VL). Cell culture media and supplements were purchased from Gibco (Life Technologies, CA, USA). Total RNA was extracted from pelleted cells using the mirVana miRNA Isolation Kit (Ambion, Life Technologies). Additional information can be found in S2 File.

### miRNA and EGFP expression analysis

RT-qPCR (reverse transcription–quantitative PCR) was performed using TaqMan microRNA assays or Power SYBR Green PCR Master Mix (Life Technologies) on a ViiA7 Real-Time PCR System (Applied Biosystems, Life Technologies) to assess the expression of mature miRNAs and EGFP in each stable cell line. Data were normalized by geometric averaging of internal control genes [50]. NRQ for miRNAs and EGFP were calculated using the ΔΔCt method based on the approach described by Hellemans et al. [51]. The miRNA NRQ of each clone was normalized for transduction efficiency by division with the EGFP NRQ value. Differential expression was tested using a two-sided unpaired t-test with unequal variance, p < 0.05 was taken as significance threshold. Additional details can be found in S2 File.

### Transcriptome analysis

200–400 ng total RNA aliquots of *MIR618* cells (clones MIR618WT1-3, MIR618SNP1-3) and *MIR204* cells (clones MIR204WT4-5, MIR204SNP4-6) were submitted for analysis with the Human Transcriptome Array 2.0 (Affymetrix, CA, USA) and data normalization using RMA to AROS Applied Biotechnology (Denmark). Prefiltering was performed on the normalized data to exclude transcript clusters lacking a gene symbol or RNA accession number. Differential expression of genes between wild type and mutant cell lines was assessed by SAM v1.0 as implemented in MeV software v4.9.0 [52,53]. A two class unpaired analysis was used (group 1: WT clones, group 2: SNP clones). GSEA was performed using GSEA v2.2.0 software [54,55] using MSigDB v5.0 C5 Gene Ontology molecular function gene sets (c5.mf.v5.0.symbols.gmt) [56]. For all comparisons (group 1: WT clones, group 2: SNP clones), data was collapsed to gene symbols, with max_probe to collapse multiple probes. Gene sets of > 500 and < 15 genes were excluded. 1000 permutations based on gene sets were performed. Gene sets were called significant when FDR < 0.01 and nominal p-value < 0.001.

### Small RNA sequencing

200–400 ng total RNA aliquots of *MIR618* cells (clones MIR618WT1-3, MIR618SNP1-3) and *MIR204* cells (clones MIR204WT4-5, MIR204SNP4-6) were submitted to Biogazelle (Belgium) for small RNA sequencing. Read QC, adaptor trimming, read mapping and annotation to miRBase 20 [22] was performed at Biogazelle for isomiRs and for mature miRNAs (defined as sum of all isomiR reads mapping to a mature miRNA locus). For miRNAs, data was prefiltered with a cutoff of four reads per miRNA per sample. Normalization and differential expression analysis of the miRNA read count data was performed using the DESeq2 Bioconductor package [20]. For DESeq2 analysis of the *MIR204* cells the independent filtering option was disabled. miRNAs with Benjamini-Hochberg adjusted p < 0.1 were called significant. For isomiR distribution analysis, isomiR read counts were normalized to the total read count of the mature miRNA (sum of all isomiRs on that locus) in each sample and counts were summed per functional group. IsomiRs were grouped per 5' start sequence: the 5' canonical group contains all isomiRs that have the same 5' end as the canonical miRNA listed in miRBase 20, isomiRs with 5' change are all other isomiRs. The two-tailed unpaired t-test with unequal variance was applied to test for differences in 5’ change isomiR frequencies between genotypes, p < 0.05 was taken as significance threshold.

### RNAhybrid prediction

Hybridization of miR-204-5p and miR-204-3p to transcripts was predicted using the RNAhybrid software v2.1.2 [21]. For miRNA targets, up- and downstream flanking regions of 200 nt were used surrounding the hairpin. Prediction was done using an energy cut-off of -20 kcal/mol and a helix constraint from positions 2 to 8 [57].

### Data availability

MASTR data has been deposited at the European Genome-phenome Archive, which is hosted by the EBI and the CRG, under accession numberEGAS00001001607. Microarray data is available at Gene Expression Omnibus with accession GSE69675. Small RNA sequencing data are available at Sequence Read Archive with study accession SRP059166.

## Supporting Information

S1 File Supplementary Strategy and Results. This file contains additional details of the variant screening strategy and additional results (Tables A-E and Figures A-F).
(DOC)

S2 File Supplementary Methods. This file contains additional details of the methods.
(DOCX)
