# Supplementary material for: Schizophrenia-Associated MIR204 Regulates Noncoding RNAs and Affects Neurotransmitter and Ion Channel Gene Sets
Source: PLoS One. 2015 Dec 29;10(12):e0144428. doi: 10.1371/journal.pone.0144428 (PMC4695081; doi:10.1371/journal.pone.0144428)
Supplement: S1 File — This file contains additional details of the variant screening strategy and additional results (Tables A-E and Figures A-F). (DOC) [file pone.0144428.s001.doc]

# S1. Supplementary Strategy and Results

# Variant screening

## MASTR assay and massively parallel sequencing

miRNA gene selection was based on expression in the human brain [1–5]. A MASTR assay was designed with the Multiplexer algorithm (Multiplicom, Belgium) [6,7] and optimized using fragment analysis to amplify the 289 miRNA genes and flanking regions into 10 multiplex PCR reactions (assay available upon request).

The assay was used to amplify the miRNA genes on genomic DNA samples of patients and control individuals. Patient samples were amplified separately, control samples were pooled per 7 samples prior to PCR amplification. Barcodes were incorporated into the PCR fragments by a second, universal, PCR step using MID for Illumina MiSeq kits (Multiplicom). To remove aspecific products and primers, barcoded samples were purified using Agencourt AMPure XP beads (Beckman Coulter, CA, USA) in a sample:beads ratio of 1:0.8. After quality assessment on LabChip GX (PerkinElmer, MA, USA), individual barcoded samples were pooled equimolarly, after which a second purification (1:0.85) was performed if necessary. 6-11pM of the final libraries (+ 1% PhiX) were subjected to sequencing on the Miseq v3 platform (Illumina, CA, USA) using 2x250 or 2x300 bp sequencing chemistry and custom sequencing primers (MID for Illumina MiSeq kits, Multiplicom) in three sequencing runs.

## Read mapping and variant calling

After adaptor clipping with fastq-mcf (<http://code.google.com/p/ea-utils>), reads were mapped to the human genome (hg 19) with BWA-MEM [8]. BAM files were realigned around indels with GATK [9]. For patient data, variant calling was performed with GATK and SAMtools [10]. For pooled control data, variant allele frequencies were calculated using SAMtools mpileup for the genomic positions of variants identified in the patient samples. Variant annotation with miRBase 20 [11] and primer sequences was performed with GenomeComb [12]. Variants in primer sequences were removed.

## Variant prioritization

Variant prioritization was performed with GenomeComb [12]. For patient data, we calculated variant frequencies and total variant allele counts per phenotype (schizophrenia, IGE), taking into account only those samples that had on the specific variant position a coverage >=20x and quality >=30 (filter step 1). To eliminate the possibility that variants where a large number of samples did not fulfill this requirement would be included, we additionally included the criterion that for variant inclusion, the position had to fulfil the coverage and quality criteria in at least 95% of the patient samples per phenotype (filter step 2). These variant frequencies and counts were calculated per variant caller separately (GATK, SAMtools). Furthermore, we required that variants included for further analysis would have the same variant frequency between GATK and SAMtools (filter step 2). As control samples were pooled, we could not apply the same analysis. Average variant frequencies were calculated based on the variant frequency per pool, only including those pools that have a coverage >=70x on the variant position (i.e. >=5x per individual allele in pool) (filter step 1). At least 50% of all pools needed to fulfil this criterion and variants needed to be present in the patient variant data set after filter step 2 to ensure high quality and coverage (filter step 2). After this prioritization, 265 high quality variants remained in the schizophrenia patients vs Swedish control group and 315 variants in the IGE patients vs Belgian/Dutch control group (Tables A and B), with an average of 45 different variants per patient sample (Figure A).

For all variants that fulfilled the criteria, we tested association with a two-sided Fisher’s exact test using the function fisher.test in R (<http://www.R-project.org/>), comparing schizophrenia samples to Swedish controls and IGE samples to Belgian/Dutch controls. Allele counts of the control group were calculated by rounding the multiplication of the calculated variant frequency with the total amount of control alleles present in the pools that fulfilled the coverage criterion (number of passed pools times 14 alleles per pool). For patients, GATK allele counts were used.

As our aim was to identify variants in miRNA genes that may regulate neuronal functions, we focused on those variants showing an altered frequency between patients and control individuals (using unadjusted p < 0.05). Ten variants had a difference in variant frequency in the schizophrenia patients versus control individuals (unadjusted p-value < 0.05) (Table C). Eight variants had a difference in variant frequency between the IGE patients and the control group (Table D). To determine whether variants were significantly associated after correction for multiple testing, we also calculated the Bonferroni adjusted p-values for all variants by multiplying the p-value with the number of tested variants per phenotype. For the variants with unadjusted p < 0.05, we assessed the variant impact by its location in the miRNA and its predicted structural impact by using miRVaS [13]. We assessed different up- and downstream flanking regions: 50, 100, 150, 200 nt and compared MFE, centroid and MEA structure predictions.

**Table A. Variants identified in the schizophrenia (SZ) patients and Swedish controls.**

| **Genomic location** | **Ref** | **Alt** | **SNP id** | **Freq (SZ)** | **Freq (CO)** | **Alt (SZ)** | **Ref (SZ)** | **Alt (CO)** | **Ref (CO)** | **P** | **Padj** |
| --- | --- | --- | --- | --- | --- | --- | --- | --- | --- | --- | --- |
| chr1:1103283-1103284 | C | T | rs202051309 | 1.08 | 0.45 | 4 | 368 | 9 | 2063 | 0.1224 | 32.45 |
| chr1:1104417-1104418 | A | C | rs200237515 | 0.54 | 0.07 | 2 | 370 | 1 | 2085 | 0.0617 | 16.34 |
| chr1:68649257-68649258 | C | T | rs147113488 | 1.08 | 0.55 | 4 | 366 | 11 | 2075 | 0.2645 | 70.09 |
| chr1:68649290-68649291 | G | C | - | 0.27 | 0.00 | 1 | 369 | 0 | 2086 | 0.1507 | 39.92 |
| chr1:68649337-68649338 | T | A | rs148820010 | 1.08 | 0.50 | 4 | 366 | 10 | 2076 | 0.1477 | 39.13 |
| chr1:71533286-71533287 | A | T | rs66461782 | 30.00 | 27.15 | 111 | 259 | 566 | 1520 | 0.2564 | 67.94 |
| chr1:71533337-71533338 | C | T | - | 0.54 | 0.20 | 2 | 368 | 4 | 2082 | 0.2249 | 59.61 |
| chr1:94312565-94312566 | G | T | rs2391318 | 100.00 | 98.49 | 372 | 0 | 2041 | 31 | 0.0100 | 2.64 |
| chr1:94312566-94312567 | T | C | rs2391319 | 100.00 | 98.66 | 372 | 0 | 2044 | 28 | 0.0155 | 4.10 |
| chr1:98511860-98511861 | A | G | rs145228502 | 0.28 | 0.44 | 1 | 359 | 8 | 1812 | 1.0000 | 265.00 |
| chr1:110141420-110141421 | T | C | rs1889470 | 17.03 | 15.15 | 63 | 307 | 316 | 1770 | 0.3496 | 92.65 |
| chr1:110141606-110141607 | G | A | - | 0.54 | 0.41 | 2 | 368 | 9 | 2077 | 0.6760 | 179.13 |
| chr1:110141681-110141682 | T | C | rs111351338 | 0.81 | 0.98 | 3 | 367 | 20 | 2066 | 1.0000 | 265.00 |
| chr1:172113791-172113792 | A | G | - | 0.27 | 0.31 | 1 | 369 | 6 | 2080 | 1.0000 | 265.00 |
| chr1:176998489-176998490 | G | T | - | 0.28 | 0.00 | 1 | 357 | 0 | 1484 | 0.1944 | 51.50 |
| chr1:193105624-193105625 | G | T | - | 0.81 | 0.44 | 3 | 367 | 9 | 2077 | 0.4064 | 107.71 |
| chr1:207975314-207975315 | C | T | rs150749580 | 0.54 | 1.17 | 2 | 368 | 24 | 2062 | 0.4120 | 109.19 |
| chr1:207975904-207975905 | C | T | rs78876157 | 0.54 | 0.48 | 2 | 368 | 10 | 2062 | 0.7015 | 185.90 |
| chr1:220291486-220291487 | T | C | rs3820455 | 5.95 | 4.80 | 22 | 348 | 100 | 1986 | 0.3627 | 96.12 |
| chr2:56210139-56210140 | G | A | rs41291173 | 2.70 | 2.59 | 10 | 360 | 53 | 2005 | 0.8592 | 227.70 |
| chr2:56210278-56210279 | T | A | rs41291175 | 5.14 | 3.60 | 19 | 351 | 75 | 1997 | 0.1850 | 49.02 |
| chr2:56210397-56210398 | A | G | rs41291177 | 22.70 | 24.26 | 84 | 286 | 499 | 1559 | 0.5522 | 146.35 |
| chr2:56216051-56216052 | G | A | rs10865292 | 89.46 | 88.70 | 331 | 39 | 1825 | 233 | 0.7206 | 190.95 |
| chr2:56216089-56216090 | A | T | rs41291179 | 5.14 | 3.75 | 19 | 351 | 77 | 1981 | 0.1948 | 51.62 |
| chr2:56216319-56216320 | C | A | rs11903947 | 21.35 | 18.12 | 79 | 291 | 373 | 1685 | 0.1470 | 38.95 |
| chr2:56227775-56227776 | G | T | rs76279789 | 5.14 | 3.80 | 19 | 351 | 79 | 2007 | 0.2473 | 65.54 |
| chr2:56227776-56227777 | C | T | rs115653519 | 5.14 | 3.95 | 19 | 351 | 82 | 2004 | 0.3185 | 84.41 |
| chr2:180725399-180725400 | C | A | rs59293459 | 11.29 | 12.76 | 42 | 330 | 261 | 1783 | 0.4958 | 131.39 |
| chr2:180725480-180725481 | T | C | rs259816 | 100.00 | 99.81 | 372 | 0 | 2054 | 4 | 1.0000 | 265.00 |
| chr2:180725567-180725568 | T | C | rs146754630 | 2.15 | 2.41 | 8 | 364 | 50 | 2008 | 0.8551 | 226.60 |
| chr2:180725700-180725701 | G | A | - | 0.27 | 0.38 | 1 | 371 | 8 | 2036 | 1.0000 | 265.00 |
| chr2:219267233-219267234 | T | C | rs144071139 | 0.28 | 0.39 | 1 | 359 | 7 | 1771 | 1.0000 | 265.00 |
| chr2:219866335-219866336 | G | C | rs6715345 | 1.08 | 1.69 | 4 | 368 | 35 | 2051 | 0.5030 | 133.29 |
| chr3:10436053-10436054 | C | G | rs73814209 | 0.54 | 1.57 | 2 | 368 | 33 | 2053 | 0.1529 | 40.53 |
| chr3:44155857-44155858 | G | A | - | 0.27 | 0.13 | 1 | 369 | 3 | 2055 | 0.4841 | 128.28 |
| chr3:47891035-47891036 | C | T | rs9871162 | 1.08 | 1.71 | 4 | 366 | 36 | 2050 | 0.5040 | 133.56 |
| chr3:49057544-49057545 | T | C | rs377407970 | 0.27 | 0.19 | 1 | 369 | 4 | 2082 | 0.5583 | 147.95 |
| chr3:49057546-49057547 | C | G | rs141806710 | 1.35 | 1.47 | 5 | 365 | 31 | 2055 | 1.0000 | 265.00 |
| chr3:49058196-49058197 | C | G | rs144723005 | 2.75 | 1.36 | 10 | 354 | 28 | 2058 | 0.0618 | 16.37 |
| chr3:160122345-160122346 | A | G | rs142685894 | 1.62 | 0.83 | 6 | 364 | 17 | 2069 | 0.1427 | 37.82 |
| chr3:160122457-160122458 | A | G | rs369598613 | 0.54 | 0.17 | 2 | 368 | 3 | 2083 | 0.1658 | 43.95 |
| chr3:160122503-160122504 | G | A | rs370054586 | 0.54 | 0.52 | 2 | 368 | 11 | 2075 | 1.0000 | 265.00 |
| chr3:168269755-168269756 | A | G | rs6771018 | 23.51 | 21.59 | 87 | 283 | 450 | 1636 | 0.4130 | 109.46 |
| chr3:168269802-168269803 | A | G | rs79926880 | 1.35 | 1.20 | 5 | 365 | 25 | 2061 | 0.7967 | 211.12 |
| chr3:186504406-186504407 | A | G | rs11538612 | 0.27 | 0.31 | 1 | 369 | 6 | 2080 | 1.0000 | 265.00 |
| chr3:186504438-186504439 | C | T | rs181852973 | 1.08 | 1.11 | 4 | 366 | 23 | 2063 | 1.0000 | 265.00 |
| chr4:8006927-8006928 | C | T | rs11939078 | 31.89 | 30.36 | 118 | 252 | 633 | 1453 | 0.5817 | 154.15 |
| chr4:8006987-8006988 | T | C | rs77249161 | 14.59 | 16.32 | 54 | 316 | 340 | 1746 | 0.4426 | 117.29 |
| chr4:8007149-8007150 | A | G | rs181064905 | 2.70 | 1.76 | 10 | 360 | 37 | 2049 | 0.2192 | 58.09 |
| chr4:8007156-8007157 | G | A | rs186841404 | 1.89 | 0.94 | 7 | 363 | 20 | 2066 | 0.1686 | 44.68 |
| chr4:115577886-115577887 | G | T | - | 0.54 | 0.22 | 2 | 368 | 4 | 2082 | 0.2249 | 59.61 |
| chr4:115577996-115577997 | C | G | rs34115976 | 20.00 | 16.65 | 74 | 296 | 347 | 1739 | 0.1163 | 30.82 |
| chr5:58999417-58999418 | C | T | - | 0.27 | 0.13 | 1 | 369 | 3 | 2055 | 0.4841 | 128.28 |
| chr5:136983244-136983245 | G | A | rs115189656 | 0.54 | 0.65 | 2 | 368 | 14 | 2072 | 1.0000 | 265.00 |
| chr5:148808389-148808390 | G | A | rs41291957 | 15.68 | 15.52 | 58 | 312 | 324 | 1762 | 0.9380 | 248.58 |
| chr5:148808473-148808474 | C | T | rs13158382 | 2.43 | 2.08 | 9 | 361 | 43 | 2043 | 0.6939 | 183.89 |
| chr5:149112299-149112300 | T | G | rs41287082 | 14.05 | 12.32 | 52 | 318 | 257 | 1829 | 0.3501 | 92.78 |
| chr5:159912417-159912418 | C | G | rs2910164 | 81.89 | 85.23 | 303 | 67 | 1778 | 308 | 0.1001 | 26.53 |
| chr5:159912503-159912504 | C | A | rs185734996 | 0.54 | 0.31 | 2 | 368 | 7 | 2079 | 0.6323 | 167.57 |
| chr5:168195143-168195144 | G | A | rs374297906 | 0.27 | 0.21 | 1 | 363 | 4 | 2082 | 0.5529 | 146.51 |
| chr5:179225323-179225324 | G | A | rs2291418 | 2.96 | 3.15 | 11 | 361 | 65 | 2007 | 1.0000 | 265.00 |
| chr5:179225479-179225480 | G | A | rs2291417 | 2.96 | 3.26 | 11 | 361 | 68 | 2004 | 0.8738 | 231.55 |
| chr6:52009245-52009246 | C | T | - | 0.54 | 0.29 | 2 | 368 | 6 | 2080 | 0.3449 | 91.40 |
| chr6:72113221-72113222 | C | T | rs72923452 | 1.38 | 0.94 | 5 | 357 | 19 | 2039 | 0.3897 | 103.28 |
| chr7:1062527-1062528 | C | T | rs1057561 | 6.45 | 6.62 | 24 | 348 | 136 | 1922 | 1.0000 | 265.00 |
| chr7:1062617-1062618 | C | T | - | 0.27 | 0.13 | 1 | 371 | 3 | 2083 | 0.4815 | 127.60 |
| chr7:1062639-1062640 | G | A | rs376453121 | 0.81 | 0.60 | 3 | 369 | 13 | 2073 | 0.7230 | 191.60 |
| chr7:1062738-1062739 | G | C | rs149150570 | 0.27 | 0.09 | 1 | 371 | 2 | 2084 | 0.3889 | 103.06 |
| chr7:1062795-1062796 | T | C | rs113664658 | 1.34 | 0.42 | 5 | 367 | 9 | 2049 | 0.0499 | 13.23 |
| chr7:5535463-5535464 | G | A | rs368679861 | 0.54 | 0.47 | 2 | 368 | 10 | 2076 | 0.6999 | 185.46 |
| chr7:5535468-5535469 | G | A | rs372099307 | 0.54 | 0.19 | 2 | 368 | 4 | 2082 | 0.2249 | 59.61 |
| chr7:99691392-99691393 | G | A | rs372015556 | 0.27 | 0.19 | 1 | 365 | 4 | 2068 | 0.5569 | 147.59 |
| chr7:126698185-126698186 | G | A | - | 0.27 | 0.13 | 1 | 369 | 3 | 2083 | 0.4798 | 127.15 |
| chr7:129410143-129410144 | G | A | rs4467881 | 58.38 | 53.31 | 216 | 154 | 1112 | 974 | 0.0792 | 21.00 |
| chr7:129410194-129410195 | G | A | rs4541843 | 54.59 | 49.33 | 202 | 168 | 1029 | 1057 | 0.0629 | 16.67 |
| chr7:129410206-129410207 | G | A | - | 0.27 | 0.45 | 1 | 369 | 9 | 2077 | 1.0000 | 265.00 |
| chr7:129410226-129410227 | C | T | rs76481776 | 8.92 | 11.08 | 33 | 337 | 231 | 1855 | 0.2370 | 62.82 |
| chr7:129410238-129410239 | C | T | rs80041074 | 0.54 | 0.90 | 2 | 368 | 19 | 2067 | 0.7584 | 200.98 |
| chr7:136587876-136587877 | C | T | - | 0.81 | 0.78 | 3 | 367 | 16 | 2070 | 1.0000 | 265.00 |
| chr7:136588079-136588080 | G | C | rs147454417 | 0.27 | 0.00 | 1 | 369 | 0 | 2086 | 0.1507 | 39.92 |
| chr7:136588108-136588109 | G | A | rs6963819 | 66.49 | 64.01 | 246 | 124 | 1335 | 751 | 0.3772 | 99.96 |
| chr8:22102518-22102519 | G | A | rs200301891 | 0.27 | 0.19 | 1 | 371 | 4 | 2082 | 0.5601 | 148.43 |
| chr8:22102682-22102683 | T | G | rs79812935 | 8.60 | 7.36 | 32 | 340 | 151 | 1907 | 0.3934 | 104.25 |
| chr8:41517930-41517931 | C | T | - | 0.27 | 0.33 | 1 | 369 | 7 | 2079 | 1.0000 | 265.00 |
| chr8:41518214-41518215 | C | T | rs187597357 | 0.27 | 0.26 | 1 | 369 | 6 | 2080 | 1.0000 | 265.00 |
| chr8:135812875-135812876 | T | G | rs41272391 | 15.93 | 14.45 | 58 | 306 | 295 | 1749 | 0.4691 | 124.32 |
| chr8:135817226-135817227 | A | G | - | 0.27 | 0.19 | 1 | 369 | 4 | 2082 | 0.5583 | 147.95 |
| chr9:4850420-4850421 | C | G | rs455864 | 90.76 | 90.34 | 334 | 34 | 1884 | 202 | 0.8483 | 224.79 |
| chr9:20716249-20716250 | A | G | rs79816418 | 2.97 | 3.26 | 11 | 359 | 68 | 2018 | 0.8738 | 231.57 |
| chr9:21512285-21512286 | C | T | rs149347978 | 0.27 | 0.57 | 1 | 369 | 12 | 2074 | 0.7055 | 186.94 |
| chr9:28863749-28863750 | C | T | rs116960285 | 1.62 | 2.34 | 6 | 364 | 49 | 2037 | 0.4513 | 119.58 |
| chr9:73424783-73424784 | C | T | rs7861254 | 16.39 | 25.34 | 60 | 306 | 529 | 1557 | 0.0002 | 0.04 |
| chr9:96938380-96938381 | A | G | - | 0.27 | 0.04 | 1 | 367 | 1 | 2085 | 0.2775 | 73.53 |
| chr9:97847881-97847882 | G | A | - | 0.55 | 1.15 | 2 | 360 | 24 | 2048 | 0.4123 | 109.25 |
| chr9:97848150-97848151 | C | T | - | 0.27 | 0.72 | 1 | 363 | 15 | 2043 | 0.4925 | 130.50 |
| chr9:97848174-97848175 | T | G | - | 1.10 | 0.44 | 4 | 360 | 9 | 2063 | 0.1162 | 30.78 |
| chr9:97848243-97848244 | G | A | - | 0.27 | 0.08 | 1 | 363 | 2 | 2084 | 0.3829 | 101.47 |
| chr9:116971621-116971622 | G | C | rs2060133 | 9.41 | 9.71 | 35 | 337 | 203 | 1883 | 0.9242 | 244.92 |
| chr9:116971708-116971709 | G | A | rs7026363 | 3.49 | 3.16 | 13 | 359 | 66 | 2020 | 0.7492 | 198.55 |
| chr9:127454605-127454606 | A | T | rs77418916 | 0.27 | 0.15 | 1 | 369 | 3 | 2083 | 0.4798 | 127.15 |
| chr9:131155026-131155027 | G | T | rs78202609 | 3.23 | 4.64 | 12 | 360 | 94 | 1936 | 0.2719 | 72.05 |
| chr9:139565284-139565285 | C | T | rs1140713 | 12.78 | 12.26 | 46 | 314 | 240 | 1720 | 0.7936 | 210.30 |
| chr10:88024442-88024443 | T | C | rs2607863 | 93.24 | 95.43 | 345 | 25 | 1991 | 95 | 0.0875 | 23.18 |
| chr10:91352331-91352332 | T | C | - | 0.27 | 0.05 | 1 | 369 | 1 | 2085 | 0.2787 | 73.84 |
| chr10:91352379-91352380 | A | T | - | 0.27 | 0.10 | 1 | 369 | 2 | 2084 | 0.3874 | 102.67 |
| chr10:105154088-105154089 | A | G | rs7911488 | 34.05 | 31.25 | 126 | 244 | 652 | 1434 | 0.3027 | 80.22 |
| chr11:2155520-2155521 | C | T | rs11564729 | 2.16 | 1.48 | 8 | 362 | 28 | 1862 | 0.3611 | 95.70 |
| chr11:64658629-64658630 | C | A | - | 0.27 | 0.12 | 1 | 369 | 2 | 2084 | 0.3874 | 102.67 |
| chr11:64658940-64658941 | G | A | - | 0.27 | 0.17 | 1 | 369 | 4 | 2082 | 0.5583 | 147.95 |
| chr11:64659000-64659001 | T | C | - | 0.27 | 0.17 | 1 | 369 | 4 | 2082 | 0.5583 | 147.95 |
| chr11:122022926-122022927 | C | T | rs543412 | 30.94 | 28.75 | 112 | 250 | 588 | 1456 | 0.4146 | 109.87 |
| chr12:7073217-7073218 | G | A | rs41279088 | 0.54 | 0.78 | 2 | 368 | 16 | 2056 | 1.0000 | 265.00 |
| chr12:7073371-7073372 | A | G | rs368910565 | 0.27 | 0.80 | 1 | 369 | 17 | 2055 | 0.5037 | 133.48 |
| chr12:49048283-49048284 | G | A | - | 0.27 | 0.18 | 1 | 369 | 4 | 2082 | 0.5583 | 147.95 |
| chr12:49048505-49048506 | C | T | rs17238458 | 2.70 | 5.08 | 10 | 360 | 106 | 1980 | 0.0461 | 12.22 |
| chr12:58218381-58218382 | G | A | rs41292017 | 2.19 | 1.55 | 8 | 358 | 32 | 2054 | 0.3691 | 97.82 |
| chr12:79812960-79812961 | A | G | - | 0.54 | 0.15 | 2 | 368 | 3 | 2083 | 0.1658 | 43.95 |
| chr12:81329535-81329536 | A | C | rs2682818 | 85.41 | 86.13 | 316 | 54 | 1797 | 289 | 0.6848 | 181.47 |
| chr12:81329671-81329672 | A | G | rs11613504 | 4.86 | 5.33 | 18 | 352 | 111 | 1975 | 0.8010 | 212.26 |
| chr12:81329736-81329737 | A | G | rs17732904 | 0.54 | 1.21 | 2 | 368 | 25 | 2061 | 0.4149 | 109.94 |
| chr12:97885495-97885496 | T | C | rs61943698 | 1.89 | 1.83 | 7 | 363 | 38 | 2048 | 0.8355 | 221.40 |
| chr12:97957701-97957702 | G | C | rs373390757 | 0.27 | 0.01 | 1 | 363 | 0 | 2044 | 0.1512 | 40.06 |
| chr13:50623476-50623477 | T | C | rs41284812 | 2.78 | 1.65 | 10 | 350 | 34 | 2024 | 0.1376 | 36.46 |
| chr13:50623536-50623537 | C | G | rs41284814 | 2.78 | 1.60 | 10 | 350 | 33 | 2025 | 0.1290 | 34.20 |
| chr13:92002804-92002805 | A | G | rs373341333 | 0.27 | 0.99 | 1 | 369 | 20 | 2038 | 0.2342 | 62.07 |
| chr13:92003509-92003510 | A | G | - | 0.27 | 0.20 | 1 | 369 | 4 | 2082 | 0.5583 | 147.95 |
| chr14:100575885-100575886 | A | G | - | 0.54 | 0.18 | 2 | 366 | 4 | 2082 | 0.2233 | 59.17 |
| chr14:100774202-100774203 | C | T | rs72631832 | 2.70 | 2.41 | 10 | 360 | 50 | 2036 | 0.7147 | 189.38 |
| chr14:101318836-101318837 | A | G | rs113399730 | 0.27 | 0.38 | 1 | 369 | 8 | 2078 | 1.0000 | 265.00 |
| chr14:101335603-101335604 | A | C | rs12886011 | 35.95 | 37.66 | 133 | 237 | 422 | 698 | 0.5770 | 152.89 |
| chr14:101348184-101348185 | C | G | - | 0.27 | 0.05 | 1 | 369 | 1 | 2085 | 0.2787 | 73.84 |
| chr14:101350934-101350935 | T | C | rs182315655 | 0.81 | 0.79 | 3 | 367 | 16 | 2070 | 1.0000 | 265.00 |
| chr14:101488532-101488533 | G | A | rs8011965 | 0.54 | 0.14 | 2 | 368 | 3 | 2069 | 0.1674 | 44.37 |
| chr14:101489702-101489703 | T | C | rs111906529 | 1.62 | 2.73 | 6 | 364 | 57 | 2029 | 0.2831 | 75.02 |
| chr14:101489857-101489858 | C | T | rs12892719 | 30.54 | 28.10 | 113 | 257 | 586 | 1500 | 0.3485 | 92.36 |
| chr14:101491374-101491375 | T | C | - | 0.81 | 0.95 | 3 | 367 | 20 | 2066 | 1.0000 | 265.00 |
| chr14:101491836-101491837 | T | G | - | 0.27 | 0.16 | 1 | 369 | 3 | 2083 | 0.4798 | 127.15 |
| chr14:101496122-101496123 | G | A | rs143863696 | 2.43 | 2.61 | 9 | 361 | 54 | 2032 | 1.0000 | 265.00 |
| chr14:101498231-101498232 | C | T | rs61992660 | 4.32 | 3.01 | 16 | 354 | 63 | 2023 | 0.2000 | 52.99 |
| chr14:101498286-101498287 | G | A | rs187860444 | 0.27 | 0.15 | 1 | 369 | 3 | 2083 | 0.4798 | 127.15 |
| chr14:101498314-101498315 | G | C | rs118116043 | 0.81 | 0.37 | 3 | 367 | 8 | 2078 | 0.2229 | 59.06 |
| chr14:101499988-101499989 | T | A | rs142673707 | 0.54 | 0.83 | 2 | 368 | 17 | 2069 | 0.7560 | 200.34 |
| chr14:101500026-101500027 | C | T | rs113479942 | 2.70 | 3.90 | 10 | 360 | 81 | 2005 | 0.2995 | 79.36 |
| chr14:101506009-101506010 | A | G | rs117381856 | 1.89 | 0.92 | 7 | 363 | 19 | 2067 | 0.0978 | 25.91 |
| chr14:101506164-101506165 | C | A | - | 0.54 | 0.34 | 2 | 368 | 7 | 2079 | 0.6323 | 167.57 |
| chr14:101506285-101506286 | A | G | - | 0.81 | 0.97 | 3 | 367 | 20 | 2066 | 1.0000 | 265.00 |
| chr14:101506440-101506441 | G | A | rs373534314 | 0.27 | 0.11 | 1 | 369 | 2 | 2084 | 0.3874 | 102.67 |
| chr14:101506532-101506533 | G | T | rs72700572 | 1.89 | 1.72 | 7 | 363 | 36 | 2050 | 0.8292 | 219.73 |
| chr14:101506554-101506555 | A | G | rs76847254 | 7.57 | 8.28 | 28 | 342 | 173 | 1913 | 0.6820 | 180.72 |
| chr14:101509224-101509225 | C | G | rs75777456 | 1.89 | 2.85 | 7 | 363 | 59 | 2013 | 0.3839 | 101.73 |
| chr14:101509269-101509270 | C | T | rs112910713 | 2.70 | 3.97 | 10 | 360 | 82 | 1990 | 0.2993 | 79.32 |
| chr14:101509273-101509274 | G | A | rs112829040 | 1.89 | 2.95 | 7 | 363 | 61 | 2011 | 0.3059 | 81.06 |
| chr14:101509286-101509287 | A | G | rs7147329 | 8.11 | 7.07 | 30 | 340 | 146 | 1926 | 0.4463 | 118.27 |
| chr14:101509448-101509449 | G | A | rs189513510 | 0.81 | 1.03 | 3 | 367 | 21 | 2051 | 1.0000 | 265.00 |
| chr14:101510399-101510400 | G | T | rs142240732 | 0.81 | 0.32 | 3 | 367 | 7 | 2079 | 0.1812 | 48.01 |
| chr14:101513794-101513795 | C | T | rs72700576 | 4.05 | 4.57 | 15 | 355 | 95 | 1991 | 0.7852 | 208.07 |
| chr14:101513799-101513800 | G | A | rs76250615 | 0.27 | 0.59 | 1 | 369 | 12 | 2074 | 0.7055 | 186.94 |
| chr14:101513902-101513903 | C | T | rs148158663 | 0.27 | 0.46 | 1 | 369 | 10 | 2076 | 1.0000 | 265.00 |
| chr14:101514360-101514361 | G | A | rs183941536 | 0.27 | 0.85 | 1 | 369 | 18 | 2068 | 0.3409 | 90.33 |
| chr14:101514370-101514371 | T | C | rs10132916 | 30.27 | 30.22 | 112 | 258 | 630 | 1456 | 1.0000 | 265.00 |
| chr14:101518759-101518760 | G | A | rs1951032 | 18.65 | 18.41 | 69 | 301 | 384 | 1702 | 0.9420 | 249.64 |
| chr14:101518893-101518894 | G | A | rs77603994 | 0.54 | 0.34 | 2 | 368 | 7 | 2079 | 0.6323 | 167.57 |
| chr14:101518919-101518920 | G | A | rs74573280 | 0.27 | 0.55 | 1 | 369 | 12 | 2074 | 0.7055 | 186.94 |
| chr14:101520526-101520527 | A | C | rs28752084 | 19.19 | 19.89 | 71 | 299 | 415 | 1671 | 0.7775 | 206.05 |
| chr14:101521151-101521152 | C | G | rs118127537 | 1.08 | 1.92 | 4 | 366 | 40 | 2032 | 0.3932 | 104.19 |
| chr14:101521703-101521704 | G | A | rs79064704 | 0.27 | 0.61 | 1 | 369 | 13 | 2073 | 0.7081 | 187.65 |
| chr14:101522555-101522556 | T | C | rs56103835 | 18.92 | 19.35 | 70 | 300 | 404 | 1682 | 0.8864 | 234.91 |
| chr14:101522670-101522671 | G | A | - | 0.27 | 0.11 | 1 | 369 | 2 | 2084 | 0.3874 | 102.67 |
| chr14:101526078-101526079 | T | G | rs77622534 | 0.27 | 0.50 | 1 | 369 | 10 | 2076 | 1.0000 | 265.00 |
| chr14:101526180-101526181 | G | A | rs41286572 | 10.81 | 10.34 | 40 | 330 | 216 | 1870 | 0.7821 | 207.24 |
| chr14:101527032-101527033 | C | T | rs78425563 | 0.54 | 0.59 | 2 | 368 | 12 | 2074 | 1.0000 | 265.00 |
| chr14:101527117-101527118 | C | T | rs117924003 | 2.97 | 2.10 | 11 | 359 | 44 | 2042 | 0.3377 | 89.48 |
| chr14:101528523-101528524 | C | T | rs117311466 | 1.08 | 0.95 | 4 | 366 | 20 | 2066 | 0.7748 | 205.33 |
| chr14:101528539-101528540 | C | T | - | 0.54 | 0.16 | 2 | 368 | 3 | 2083 | 0.1658 | 43.95 |
| chr14:101530728-101530729 | G | A | - | 0.54 | 0.83 | 2 | 368 | 17 | 2041 | 0.7556 | 200.24 |
| chr14:101530947-101530948 | G | A | rs370573398 | 1.35 | 0.83 | 5 | 365 | 17 | 2041 | 0.3648 | 96.68 |
| chr14:101531567-101531568 | G | C | rs150699693 | 1.35 | 1.68 | 5 | 365 | 35 | 2051 | 0.8244 | 218.47 |
| chr14:101531693-101531694 | G | A | rs368490724 | 0.27 | 0.20 | 1 | 369 | 4 | 2082 | 0.5583 | 147.95 |
| chr14:101531732-101531733 | C | T | rs61992670 | 4.59 | 3.89 | 17 | 353 | 81 | 2005 | 0.4745 | 125.76 |
| chr14:101531856-101531857 | C | T | rs139967426 | 0.27 | 0.65 | 1 | 369 | 14 | 2072 | 0.7140 | 189.20 |
| chr14:101531919-101531920 | C | T | rs145707596 | 0.27 | 0.61 | 1 | 369 | 13 | 2073 | 0.7081 | 187.65 |
| chr14:101532216-101532217 | G | A | rs10144831 | 5.14 | 4.98 | 19 | 351 | 104 | 1982 | 0.8971 | 237.74 |
| chr14:101532235-101532236 | C | T | rs10144688 | 0.27 | 0.32 | 1 | 369 | 7 | 2079 | 1.0000 | 265.00 |
| chr14:101532901-101532902 | C | T | - | 0.27 | 0.14 | 1 | 369 | 3 | 2083 | 0.4798 | 127.15 |
| chr14:101533092-101533093 | C | T | rs58834075 | 4.05 | 3.44 | 15 | 355 | 72 | 2014 | 0.5424 | 143.74 |
| chr14:102026767-102026768 | G | C | - | 0.54 | 0.27 | 2 | 370 | 6 | 2052 | 0.3526 | 93.45 |
| chr15:55665088-55665089 | T | C | rs8041885 | 8.65 | 9.38 | 32 | 338 | 196 | 1890 | 0.6982 | 185.03 |
| chr15:55665312-55665313 | C | A | rs8041044 | 8.65 | 9.32 | 32 | 338 | 194 | 1892 | 0.7698 | 204.00 |
| chr15:70371776-70371777 | C | G | rs78212770 | 1.62 | 1.25 | 6 | 364 | 26 | 2046 | 0.6171 | 163.52 |
| chr15:70371829-70371830 | C | T | rs148893826 | 0.54 | 0.71 | 2 | 368 | 15 | 2057 | 1.0000 | 265.00 |
| chr15:70371866-70371867 | C | T | rs79128009 | 2.16 | 1.85 | 8 | 362 | 38 | 2034 | 0.6771 | 179.42 |
| chr15:70371873-70371874 | G | C | rs75165322 | 2.16 | 1.95 | 8 | 362 | 40 | 2032 | 0.6879 | 182.29 |
| chr15:89151215-89151216 | T | C | - | 0.27 | 0.11 | 1 | 369 | 2 | 2056 | 0.3912 | 103.66 |
| chr15:89154965-89154966 | G | A | rs7170666 | 3.51 | 4.00 | 13 | 357 | 84 | 2002 | 0.7720 | 204.59 |
| chr15:89155072-89155073 | C | T | rs41276930 | 1.08 | 0.62 | 4 | 366 | 13 | 2073 | 0.3081 | 81.66 |
| chr15:89911354-89911355 | C | G | - | 0.27 | 0.01 | 1 | 371 | 0 | 2072 | 0.1522 | 40.34 |
| chr16:2321826-2321827 | C | T | rs373106490 | 0.27 | 0.26 | 1 | 371 | 5 | 2039 | 1.0000 | 265.00 |
| chr16:14397923-14397924 | T | C | rs112887255 | 1.08 | 2.43 | 4 | 366 | 51 | 2035 | 0.1259 | 33.37 |
| chr16:56892554-56892555 | C | T | - | 0.27 | 0.09 | 1 | 369 | 2 | 2070 | 0.3893 | 103.16 |
| chr16:56892592-56892593 | G | A | rs76987351 | 1.08 | 2.60 | 4 | 366 | 54 | 2004 | 0.0935 | 24.77 |
| chr16:67236108-67236109 | A | T | rs192034781 | 0.81 | 0.99 | 3 | 367 | 21 | 2065 | 1.0000 | 265.00 |
| chr16:67236291-67236292 | C | G | rs188892061 | 0.54 | 0.19 | 2 | 368 | 4 | 2082 | 0.2249 | 59.61 |
| chr16:69967092-69967093 | G | A | rs2102066 | 69.73 | 70.11 | 258 | 112 | 1443 | 615 | 0.9019 | 239.00 |
| chr17:1617175-1617176 | C | T | rs373333946 | 0.54 | 0.32 | 2 | 368 | 7 | 2079 | 0.6323 | 167.57 |
| chr17:6920887-6920888 | C | T | rs41283391 | 5.71 | 5.38 | 21 | 347 | 112 | 1974 | 0.8026 | 212.70 |
| chr17:11985363-11985364 | C | G | - | 0.27 | 0.00 | 1 | 369 | 0 | 2086 | 0.1507 | 39.92 |
| chr17:28444182-28444183 | A | C | rs6505162 | 49.46 | 46.88 | 183 | 187 | 978 | 1108 | 0.3665 | 97.13 |
| chr17:46114571-46114572 | C | G | rs200114569 | 0.27 | 0.39 | 1 | 369 | 8 | 2050 | 1.0000 | 265.00 |
| chr17:46114619-46114620 | C | T | rs12940701 | 26.49 | 28.99 | 98 | 272 | 597 | 1461 | 0.3489 | 92.45 |
| chr17:46114691-46114692 | C | G | rs41280120 | 25.41 | 21.01 | 94 | 276 | 432 | 1626 | 0.0641 | 16.98 |
| chr17:57918581-57918582 | G | C | rs79800958 | 0.27 | 0.00 | 1 | 363 | 0 | 2086 | 0.1486 | 39.37 |
| chr18:19263567-19263568 | A | G | rs147020262 | 1.35 | 1.12 | 5 | 365 | 23 | 2049 | 0.6019 | 159.51 |
| chr18:19408890-19408891 | A | G | rs78641532 | 7.03 | 7.80 | 26 | 344 | 163 | 1923 | 0.6724 | 178.20 |
| chr18:19408949-19408950 | C | T | rs9989532 | 98.92 | 97.86 | 366 | 4 | 2041 | 45 | 0.2256 | 59.78 |
| chr18:33484791-33484792 | G | A | rs41274312 | 1.62 | 2.85 | 6 | 364 | 59 | 2027 | 0.2198 | 58.25 |
| chr18:33484836-33484837 | C | T | rs375688661 | 0.27 | 0.45 | 1 | 369 | 9 | 2077 | 1.0000 | 265.00 |
| chr18:56118357-56118358 | C | T | rs41292412 | 0.54 | 0.44 | 2 | 368 | 9 | 2077 | 0.6760 | 179.13 |
| chr19:10928013-10928014 | C | A | - | 0.27 | 0.01 | 1 | 369 | 0 | 2086 | 0.1507 | 39.92 |
| chr19:10928054-10928055 | C | T | - | 0.27 | 0.12 | 1 | 369 | 2 | 2084 | 0.3874 | 102.67 |
| chr19:10928062-10928063 | T | C | rs375081336 | 1.89 | 2.52 | 7 | 363 | 53 | 2033 | 0.5839 | 154.72 |
| chr19:10928080-10928081 | C | T | rs1005039 | 0.27 | 0.77 | 1 | 369 | 16 | 2070 | 0.4956 | 131.33 |
| chr19:10928178-10928179 | G | A | rs373701104 | 1.35 | 0.34 | 5 | 365 | 7 | 2079 | 0.0240 | 6.37 |
| chr19:13947295-13947296 | G | A | rs11671784 | 1.35 | 1.15 | 5 | 365 | 24 | 2062 | 0.7925 | 210.01 |
| chr19:40788441-40788442 | T | G | rs73933241 | 5.95 | 6.69 | 22 | 348 | 139 | 1933 | 0.6501 | 172.28 |
| chr19:40788476-40788477 | A | C | rs147213560 | 0.27 | 0.01 | 1 | 369 | 0 | 2072 | 0.1515 | 40.15 |
| chr19:50004169-50004170 | G | A | rs371322685 | 0.27 | 0.16 | 1 | 371 | 3 | 2083 | 0.4815 | 127.60 |
| chr19:52196253-52196254 | C | G | - | 0.56 | 0.26 | 2 | 356 | 5 | 1759 | 0.3360 | 89.03 |
| chr19:52196408-52196409 | G | A | rs41275794 | 3.06 | 1.65 | 11 | 349 | 34 | 2010 | 0.0885 | 23.44 |
| chr19:52196452-52196453 | T | C | rs12976445 | 38.89 | 36.93 | 140 | 220 | 755 | 1289 | 0.4787 | 126.86 |
| chr19:54240087-54240088 | G | A | rs77959394 | 5.41 | 5.77 | 20 | 350 | 120 | 1966 | 0.9032 | 239.35 |
| chr19:54240183-54240184 | T | A | rs151073675 | 0.27 | 0.48 | 1 | 369 | 10 | 2076 | 1.0000 | 265.00 |
| chr19:54240218-54240219 | G | A | rs141076796 | 1.35 | 1.02 | 5 | 365 | 21 | 2065 | 0.5783 | 153.25 |
| chr20:57392685-57392686 | G | A | rs117258475 | 1.35 | 1.04 | 5 | 365 | 22 | 2064 | 0.5883 | 155.89 |
| chr20:61151713-61151714 | G | A | rs74510555 | 0.54 | 0.89 | 2 | 368 | 18 | 2012 | 0.7565 | 200.47 |
| chr20:61162099-61162100 | G | A | rs13040413 | 22.16 | 21.21 | 82 | 288 | 442 | 1644 | 0.6797 | 180.12 |
| chr20:61162227-61162228 | C | T | rs199563235 | 0.27 | 0.32 | 1 | 369 | 7 | 2079 | 1.0000 | 265.00 |
| chr20:61162266-61162267 | T | C | rs6062251 | 52.16 | 49.50 | 193 | 177 | 1033 | 1053 | 0.3669 | 97.23 |
| chr20:61162288-61162289 | G | A | rs149629841 | 1.62 | 1.81 | 6 | 364 | 38 | 2048 | 1.0000 | 265.00 |
| chr21:17912241-17912242 | C | T | - | 0.27 | 0.09 | 1 | 363 | 2 | 2084 | 0.3829 | 101.47 |
| chr21:17962381-17962382 | T | A | rs2823897 | 1.37 | 3.16 | 5 | 359 | 65 | 1993 | 0.0621 | 16.46 |
| chr21:17962660-17962661 | C | T | rs143768113 | 0.82 | 0.90 | 3 | 361 | 18 | 2040 | 1.0000 | 265.00 |
| chr22:20236631-20236632 | T | C | rs9618761 | 0.27 | 0.30 | 1 | 369 | 6 | 2080 | 1.0000 | 265.00 |
| chr22:22007633-22007634 | G | T | - | 0.81 | 0.27 | 3 | 369 | 6 | 2066 | 0.1453 | 38.49 |
| chrX:49767831-49767832 | A | G | rs456615 | 100.00 | 99.89 | 370 | 0 | 2084 | 2 | 1.0000 | 265.00 |
| chrX:49767834-49767835 | A | G | rs456617 | 100.00 | 99.91 | 370 | 0 | 2084 | 2 | 1.0000 | 265.00 |
| chrX:49767903-49767904 | A | G | rs149259 | 100.00 | 99.86 | 370 | 0 | 2083 | 3 | 1.0000 | 265.00 |
| chrX:49773086-49773087 | G | A | rs191321849 | 0.54 | 1.10 | 2 | 368 | 23 | 2035 | 0.4114 | 109.03 |
| chrX:49773299-49773300 | C | T | - | 0.54 | 0.33 | 2 | 368 | 7 | 2051 | 0.6342 | 168.05 |
| chrX:49773804-49773805 | A | C | rs149596 | 100.00 | 99.92 | 370 | 0 | 2084 | 2 | 1.0000 | 265.00 |
| chrX:49774446-49774447 | C | T | rs149202543 | 5.68 | 2.35 | 21 | 349 | 49 | 2037 | 0.0011 | 0.29 |
| chrX:49775217-49775218 | G | A | - | 0.27 | 0.48 | 1 | 369 | 10 | 2076 | 1.0000 | 265.00 |
| chrX:73438223-73438224 | C | T | rs372055341 | 0.81 | 1.15 | 3 | 367 | 24 | 2062 | 0.7874 | 208.67 |
| chrX:73507209-73507210 | G | T | rs375278491 | 0.81 | 0.99 | 3 | 367 | 21 | 2065 | 1.0000 | 265.00 |
| chrX:85158799-85158800 | T | A | rs141029572 | 1.08 | 0.51 | 4 | 366 | 11 | 2075 | 0.2645 | 70.09 |
| chrX:109298666-109298667 | C | T | rs189465905 | 1.08 | 0.88 | 4 | 366 | 18 | 2054 | 0.7629 | 202.18 |
| chrX:133303757-133303758 | G | A | rs193073651 | 0.54 | 0.18 | 2 | 368 | 4 | 2082 | 0.2249 | 59.61 |
| chrX:133675340-133675341 | G | A | rs374553549 | 2.16 | 1.04 | 8 | 362 | 22 | 2050 | 0.1171 | 31.02 |
| chrX:139006270-139006271 | C | G | rs139318512 | 0.81 | 1.11 | 3 | 367 | 23 | 2063 | 0.7868 | 208.50 |
| chrX:140008384-140008385 | G | T | rs5907732 | 77.30 | 76.00 | 286 | 84 | 1585 | 501 | 0.6430 | 170.40 |
| chrX:140008438-140008439 | G | A | rs144296899 | 1.35 | 0.79 | 5 | 365 | 16 | 2070 | 0.2311 | 61.25 |
| chrX:146341310-146341311 | C | A | rs78095394 | 7.30 | 4.87 | 27 | 343 | 102 | 1984 | 0.0586 | 15.52 |
| chrX:146342027-146342028 | C | T | rs187006550 | 1.62 | 0.33 | 6 | 364 | 7 | 2065 | 0.0078 | 2.05 |
| chrX:146342232-146342233 | T | C | rs73243481 | 1.62 | 0.64 | 6 | 364 | 13 | 2059 | 0.0553 | 14.65 |
| chrX:146342250-146342251 | C | T | rs7885181 | 1.62 | 0.48 | 6 | 364 | 10 | 2062 | 0.0242 | 6.42 |
| chrX:151562021-151562022 | T | A | rs184094665 | 1.62 | 0.21 | 6 | 364 | 4 | 2082 | 0.0014 | 0.37 |

Genomic location: coordinates in zero-based half open format, hg19. Freq(SZ) (%): allele frequency of the variant in the schizophrenia patient group (%). Freq(CO): allele frequency of the variant in the group of Swedish control pools (%). Alt(SZ): number of alternative alleles in the group of schizophrenia patients. Ref(SZ): number of reference alleles in the group of schizophrenia patients. Alt(CO): number of alternative alleles in the group of Swedish control pools. Ref(CO): number of reference alleles in the group of Swedish control pools. P: Fisher’s exact p-value (rounded). Padj: Bonferroni corrected p-value (rounded).

**Table B**. Variants identified in the patients with IGE and Belgian/Dutch controls.

| **Genomic location** | **Ref** | **Alt** | **SNP id** | **Freq (IGE)** | **Freq (CO)** | **Alt (IGE)** | **Ref (IGE)** | **Alt (CO)** | **Ref (CO)** | **P** | **Padj** |
| --- | --- | --- | --- | --- | --- | --- | --- | --- | --- | --- | --- |
| chr1:1102562-1102563 | G | A | rs72563729 | 1.24 | 1.85 | 4 | 318 | 10 | 522 | 0.5856 | 184.47 |
| chr1:1102673-1102674 | G | A | rs143277733 | 0.31 | 0.24 | 1 | 321 | 1 | 545 | 1.0000 | 315.00 |
| chr1:1103235-1103236 | G | A | - | 0.31 | 0.18 | 1 | 323 | 1 | 545 | 1.0000 | 315.00 |
| chr1:1103283-1103284 | C | T | rs202051309 | 0.93 | 0.73 | 3 | 321 | 4 | 542 | 0.7157 | 225.43 |
| chr1:1103359-1103360 | A | G | rs111652490 | 0.62 | 0.27 | 2 | 322 | 1 | 545 | 0.5592 | 176.16 |
| chr1:68649257-68649258 | C | T | rs147113488 | 0.62 | 0.82 | 2 | 322 | 4 | 542 | 1.0000 | 315.00 |
| chr1:71533286-71533287 | A | T | rs66461782 | 24.07 | 21.61 | 78 | 246 | 118 | 428 | 0.4026 | 126.82 |
| chr1:71533291-71533292 | T | C | - | 0.31 | 0.12 | 1 | 323 | 1 | 545 | 1.0000 | 315.00 |
| chr1:94312565-94312566 | G | T | rs2391318 | 100.00 | 98.18 | 324 | 0 | 536 | 10 | 0.0164 | 5.16 |
| chr1:94312566-94312567 | T | C | rs2391319 | 100.00 | 98.39 | 324 | 0 | 537 | 9 | 0.0306 | 9.64 |
| chr1:110141420-110141421 | T | C | rs1889470 | 20.99 | 15.58 | 68 | 256 | 85 | 461 | 0.0528 | 16.63 |
| chr1:110141517-110141518 | T | C | - | 0.31 | 0.13 | 1 | 323 | 1 | 545 | 1.0000 | 315.00 |
| chr1:110141681-110141682 | T | C | rs111351338 | 0.31 | 0.88 | 1 | 323 | 5 | 541 | 0.4203 | 132.38 |
| chr1:156390344-156390345 | G | C | - | 0.31 | 0.00 | 1 | 323 | 0 | 546 | 0.3724 | 117.31 |
| chr1:172113791-172113792 | A | G | - | 0.62 | 0.35 | 2 | 322 | 2 | 544 | 0.6313 | 198.85 |
| chr1:176998616-176998617 | A | T | - | 0.31 | 0.12 | 1 | 323 | 1 | 545 | 1.0000 | 315.00 |
| chr1:176998705-176998706 | G | A | rs189475004 | 0.31 | 0.27 | 1 | 323 | 1 | 419 | 1.0000 | 315.00 |
| chr1:193105536-193105537 | T | A | - | 0.31 | 0.02 | 1 | 323 | 0 | 546 | 0.3724 | 117.31 |
| chr1:198828095-198828096 | A | T | rs200492369 | 0.31 | 0.03 | 1 | 323 | 0 | 546 | 0.3724 | 117.31 |
| chr1:207975314-207975315 | C | T | rs150749580 | 0.93 | 1.16 | 3 | 321 | 6 | 540 | 1.0000 | 315.00 |
| chr1:207975904-207975905 | C | T | rs78876157 | 1.54 | 1.05 | 5 | 319 | 6 | 540 | 0.5494 | 173.07 |
| chr1:220291486-220291487 | T | C | rs3820455 | 6.17 | 5.25 | 20 | 304 | 29 | 517 | 0.6488 | 204.36 |
| chr2:56210139-56210140 | G | A | rs41291173 | 2.78 | 2.08 | 9 | 315 | 11 | 535 | 0.4888 | 153.96 |
| chr2:56210278-56210279 | T | A | rs41291175 | 6.48 | 5.57 | 21 | 303 | 30 | 516 | 0.5534 | 174.33 |
| chr2:56210397-56210398 | A | G | rs41291177 | 25.31 | 24.02 | 82 | 242 | 131 | 415 | 0.6838 | 215.40 |
| chr2:56216051-56216052 | G | A | rs10865292 | 93.83 | 91.10 | 304 | 20 | 497 | 49 | 0.1545 | 48.66 |
| chr2:56216089-56216090 | A | T | rs41291179 | 6.79 | 7.02 | 22 | 302 | 38 | 508 | 1.0000 | 315.00 |
| chr2:56216246-56216247 | G | A | - | 0.31 | 0.14 | 1 | 323 | 1 | 545 | 1.0000 | 315.00 |
| chr2:56216318-56216319 | G | A | rs13431228 | 0.62 | 0.19 | 2 | 322 | 1 | 545 | 0.5592 | 176.16 |
| chr2:56216319-56216320 | C | A | rs11903947 | 24.07 | 25.53 | 78 | 246 | 139 | 407 | 0.6856 | 215.97 |
| chr2:56227775-56227776 | G | T | rs76279789 | 6.48 | 5.54 | 21 | 303 | 30 | 516 | 0.5534 | 174.33 |
| chr2:56227776-56227777 | C | T | rs115653519 | 6.48 | 5.66 | 21 | 303 | 31 | 515 | 0.6584 | 207.41 |
| chr2:56227806-56227807 | A | G | rs73940377 | 0.62 | 0.22 | 2 | 322 | 1 | 545 | 0.5592 | 176.16 |
| chr2:56227845-56227846 | A | G | rs373943940 | 0.31 | 0.16 | 1 | 323 | 1 | 545 | 1.0000 | 315.00 |
| chr2:56227890-56227891 | C | T | rs371291090 | 0.31 | 0.06 | 1 | 323 | 0 | 546 | 0.3724 | 117.31 |
| chr2:180725480-180725481 | T | C | rs259816 | 100.00 | 99.80 | 324 | 0 | 545 | 1 | 1.0000 | 315.00 |
| chr2:180725535-180725536 | C | A | rs143408979 | 0.31 | 0.15 | 1 | 323 | 1 | 545 | 1.0000 | 315.00 |
| chr2:180725567-180725568 | T | C | rs146754630 | 2.78 | 1.70 | 9 | 315 | 9 | 537 | 0.3248 | 102.31 |
| chr2:180725700-180725701 | G | A | - | 0.62 | 0.67 | 2 | 322 | 4 | 542 | 1.0000 | 315.00 |
| chr2:219267233-219267234 | T | C | rs144071139 | 0.62 | 1.50 | 2 | 322 | 7 | 469 | 0.3248 | 102.32 |
| chr2:219267306-219267307 | A | T | - | 0.31 | 0.46 | 1 | 323 | 3 | 543 | 1.0000 | 315.00 |
| chr2:219866335-219866336 | G | C | rs6715345 | 3.40 | 2.91 | 11 | 313 | 16 | 530 | 0.6910 | 217.68 |
| chr3:10436053-10436054 | C | G | rs73814209 | 3.09 | 4.34 | 10 | 314 | 24 | 522 | 0.3712 | 116.92 |
| chr3:38010963-38010964 | G | A | rs182070256 | 0.31 | 0.14 | 1 | 323 | 1 | 545 | 1.0000 | 315.00 |
| chr3:44155842-44155843 | G | C | - | 0.31 | 0.05 | 1 | 323 | 0 | 546 | 0.3724 | 117.31 |
| chr3:44155872-44155873 | C | T | - | 0.31 | 0.08 | 1 | 323 | 0 | 546 | 0.3724 | 117.31 |
| chr3:47890886-47890887 | C | T | rs74880698 | 0.93 | 0.30 | 3 | 321 | 2 | 544 | 0.3672 | 115.66 |
| chr3:47891035-47891036 | C | T | rs9871162 | 1.54 | 1.80 | 5 | 319 | 10 | 536 | 1.0000 | 315.00 |
| chr3:49057436-49057437 | C | T | - | 0.31 | 0.24 | 1 | 321 | 1 | 545 | 1.0000 | 315.00 |
| chr3:49058196-49058197 | C | G | rs144723005 | 1.85 | 1.34 | 6 | 318 | 7 | 539 | 0.5676 | 178.81 |
| chr3:160122345-160122346 | A | G | rs142685894 | 0.31 | 0.21 | 1 | 323 | 1 | 545 | 1.0000 | 315.00 |
| chr3:160122457-160122458 | A | G | rs369598613 | 0.31 | 0.05 | 1 | 323 | 0 | 546 | 0.3724 | 117.31 |
| chr3:160122503-160122504 | G | A | rs370054586 | 0.93 | 0.70 | 3 | 321 | 4 | 542 | 0.7157 | 225.43 |
| chr3:168269802-168269803 | A | G | rs79926880 | 0.62 | 1.52 | 2 | 320 | 8 | 538 | 0.3377 | 106.38 |
| chr3:186504406-186504407 | A | G | rs11538612 | 0.93 | 1.04 | 3 | 321 | 6 | 540 | 1.0000 | 315.00 |
| chr3:186504438-186504439 | C | T | rs181852973 | 1.23 | 0.39 | 4 | 320 | 2 | 544 | 0.2030 | 63.93 |
| chr4:8006927-8006928 | C | T | rs11939078 | 31.48 | 36.67 | 102 | 222 | 200 | 346 | 0.1407 | 44.30 |
| chr4:8006987-8006988 | T | C | rs77249161 | 16.67 | 14.63 | 54 | 270 | 80 | 466 | 0.4381 | 138.00 |
| chr4:8007065-8007066 | C | A | - | 0.31 | 0.04 | 1 | 323 | 0 | 546 | 0.3724 | 117.31 |
| chr4:8007156-8007157 | G | A | rs186841404 | 0.31 | 0.51 | 1 | 323 | 3 | 543 | 1.0000 | 315.00 |
| chr4:115577996-115577997 | C | G | rs34115976 | 19.44 | 16.43 | 63 | 261 | 90 | 456 | 0.2705 | 85.20 |
| chr5:136983244-136983245 | G | A | rs115189656 | 1.54 | 2.03 | 5 | 319 | 11 | 535 | 0.7956 | 250.62 |
| chr5:136983302-136983303 | T | G | - | 0.31 | 0.14 | 1 | 323 | 1 | 545 | 1.0000 | 315.00 |
| chr5:148808428-148808429 | C | T | - | 0.31 | 0.07 | 1 | 323 | 0 | 546 | 0.3724 | 117.31 |
| chr5:148808473-148808474 | C | T | rs13158382 | 2.47 | 2.89 | 8 | 316 | 16 | 530 | 0.8314 | 261.89 |
| chr5:149112299-149112300 | T | G | rs41287082 | 16.67 | 14.21 | 54 | 270 | 78 | 468 | 0.3791 | 119.43 |
| chr5:149112466-149112467 | T | G | - | 0.31 | 0.01 | 1 | 323 | 0 | 546 | 0.3724 | 117.31 |
| chr5:159912417-159912418 | C | G | rs2910164 | 73.15 | 76.83 | 237 | 87 | 419 | 127 | 0.2545 | 80.15 |
| chr5:167987880-167987881 | A | G | - | 0.31 | 0.05 | 1 | 323 | 0 | 546 | 0.3724 | 117.31 |
| chr5:168195143-168195144 | G | A | rs374297906 | 0.93 | 0.91 | 3 | 321 | 5 | 541 | 1.0000 | 315.00 |
| chr5:168195175-168195176 | G | A | rs368624931 | 0.31 | 0.13 | 1 | 323 | 1 | 545 | 1.0000 | 315.00 |
| chr5:168195207-168195208 | G | T | - | 0.31 | 0.05 | 1 | 323 | 0 | 546 | 0.3724 | 117.31 |
| chr5:179225323-179225324 | G | A | rs2291418 | 3.40 | 2.85 | 11 | 313 | 16 | 530 | 0.6910 | 217.68 |
| chr5:179225479-179225480 | G | A | rs2291417 | 3.40 | 2.88 | 11 | 313 | 16 | 530 | 0.6910 | 217.68 |
| chr5:179225498-179225499 | C | A | rs145401676 | 0.31 | 0.06 | 1 | 321 | 0 | 546 | 0.3710 | 116.85 |
| chr6:52009239-52009240 | G | A | rs369175202 | 0.31 | 0.07 | 1 | 323 | 0 | 546 | 0.3724 | 117.31 |
| chr6:72113221-72113222 | C | T | rs72923452 | 0.31 | 1.13 | 1 | 323 | 6 | 540 | 0.2677 | 84.32 |
| chr7:1062598-1062599 | T | C | rs72631820 | 0.31 | 0.93 | 1 | 323 | 5 | 541 | 0.4203 | 132.38 |
| chr7:1062738-1062739 | G | C | rs149150570 | 0.31 | 0.30 | 1 | 323 | 2 | 544 | 1.0000 | 315.00 |
| chr7:1062781-1062782 | C | T | - | 0.31 | 0.10 | 1 | 323 | 1 | 545 | 1.0000 | 315.00 |
| chr7:1062791-1062792 | C | A | rs190309950 | 0.31 | 0.00 | 1 | 323 | 0 | 546 | 0.3724 | 117.31 |
| chr7:1062795-1062796 | T | C | rs113664658 | 1.23 | 1.41 | 4 | 320 | 8 | 538 | 1.0000 | 315.00 |
| chr7:5535425-5535426 | G | A | rs192707290 | 0.62 | 0.42 | 2 | 322 | 2 | 544 | 0.6313 | 198.85 |
| chr7:5535463-5535464 | G | A | rs368679861 | 0.31 | 0.31 | 1 | 323 | 2 | 544 | 1.0000 | 315.00 |
| chr7:25989456-25989457 | G | C | - | 0.31 | 0.22 | 1 | 321 | 1 | 545 | 1.0000 | 315.00 |
| chr7:73605697-73605698 | T | C | rs372290678 | 0.31 | 0.07 | 1 | 323 | 0 | 546 | 0.3724 | 117.31 |
| chr7:99691488-99691489 | G | A | - | 0.31 | 0.13 | 1 | 323 | 1 | 545 | 1.0000 | 315.00 |
| chr7:129410226-129410227 | C | T | rs76481776 | 7.72 | 9.80 | 25 | 299 | 54 | 492 | 0.3292 | 103.69 |
| chr7:129410354-129410355 | C | T | - | 0.31 | 0.08 | 1 | 323 | 0 | 546 | 0.3724 | 117.31 |
| chr7:136588108-136588109 | G | A | rs6963819 | 61.42 | 65.66 | 199 | 125 | 358 | 188 | 0.2424 | 76.36 |
| chr7:150935760-150935761 | C | T | rs61745936 | 0.31 | 0.32 | 1 | 321 | 2 | 530 | 1.0000 | 315.00 |
| chr8:22102517-22102518 | C | T | rs373890034 | 0.31 | 0.17 | 1 | 323 | 1 | 545 | 1.0000 | 315.00 |
| chr8:22102682-22102683 | T | G | rs79812935 | 4.04 | 6.31 | 13 | 309 | 34 | 512 | 0.2140 | 67.40 |
| chr8:65291918-65291919 | C | G | rs80137356 | 0.31 | 0.21 | 1 | 323 | 1 | 531 | 1.0000 | 315.00 |
| chr8:65291929-65291930 | T | C | rs368018577 | 0.31 | 0.46 | 1 | 323 | 2 | 530 | 1.0000 | 315.00 |
| chr8:135812732-135812733 | A | T | rs371916605 | 0.93 | 0.18 | 3 | 321 | 1 | 545 | 0.1484 | 46.74 |
| chr8:135812875-135812876 | T | G | rs41272391 | 8.02 | 11.66 | 26 | 298 | 64 | 482 | 0.0855 | 26.94 |
| chr8:135812878-135812879 | G | A | - | 0.31 | 0.07 | 1 | 323 | 0 | 546 | 0.3724 | 117.31 |
| chr8:141742638-141742639 | T | C | rs373727000 | 0.62 | 0.20 | 2 | 322 | 1 | 545 | 0.5592 | 176.16 |
| chr9:4850420-4850421 | C | G | rs455864 | 89.13 | 89.67 | 287 | 35 | 490 | 56 | 0.8188 | 257.92 |
| chr9:4850435-4850436 | G | T | rs462480 | 62.73 | 56.99 | 202 | 120 | 311 | 235 | 0.1005 | 31.66 |
| chr9:20716062-20716063 | A | C | rs143382310 | 0.31 | 0.03 | 1 | 323 | 0 | 546 | 0.3724 | 117.31 |
| chr9:20716068-20716069 | T | C | rs112915556 | 0.62 | 1.03 | 2 | 322 | 6 | 540 | 0.7171 | 225.88 |
| chr9:20716249-20716250 | A | G | rs79816418 | 5.86 | 4.65 | 19 | 305 | 25 | 521 | 0.4258 | 134.12 |
| chr9:21512285-21512286 | C | T | rs149347978 | 0.93 | 0.58 | 3 | 321 | 3 | 543 | 0.6762 | 213.02 |
| chr9:28863749-28863750 | C | T | rs116960285 | 0.31 | 0.81 | 1 | 323 | 4 | 542 | 0.6562 | 206.69 |
| chr9:28863807-28863808 | T | C | - | 0.31 | 0.08 | 1 | 323 | 0 | 546 | 0.3724 | 117.31 |
| chr9:73424783-73424784 | C | T | rs7861254 | 23.46 | 21.47 | 76 | 248 | 117 | 429 | 0.5002 | 157.58 |
| chr9:97847626-97847627 | A | C | - | 0.62 | 0.06 | 2 | 322 | 0 | 546 | 0.1384 | 43.60 |
| chr9:116971621-116971622 | G | C | rs2060133 | 13.58 | 14.48 | 44 | 280 | 79 | 467 | 0.7632 | 240.40 |
| chr9:116971680-116971681 | G | A | rs189248569 | 0.93 | 0.60 | 3 | 321 | 3 | 543 | 0.6762 | 213.02 |
| chr9:116971708-116971709 | G | A | rs7026363 | 3.70 | 3.58 | 12 | 312 | 20 | 526 | 1.0000 | 315.00 |
| chr9:127454605-127454606 | A | T | rs77418916 | 0.62 | 0.25 | 2 | 322 | 1 | 545 | 0.5592 | 176.16 |
| chr9:127454655-127454656 | G | A | - | 0.31 | 0.06 | 1 | 323 | 0 | 546 | 0.3724 | 117.31 |
| chr9:127454842-127454843 | C | T | - | 0.31 | 0.05 | 1 | 323 | 0 | 546 | 0.3724 | 117.31 |
| chr9:131155026-131155027 | G | T | rs78202609 | 4.63 | 4.91 | 15 | 309 | 24 | 466 | 1.0000 | 315.00 |
| chr9:139565133-139565134 | C | T | rs199992070 | 0.31 | 0.10 | 1 | 323 | 1 | 545 | 1.0000 | 315.00 |
| chr9:139565208-139565209 | G | A | rs181698452 | 0.31 | 0.26 | 1 | 323 | 1 | 545 | 1.0000 | 315.00 |
| chr10:88024442-88024443 | T | C | rs2607863 | 95.68 | 93.71 | 310 | 14 | 512 | 34 | 0.2829 | 89.11 |
| chr10:88024552-88024553 | C | T | rs10887569 | 62.35 | 65.67 | 202 | 122 | 359 | 187 | 0.3409 | 107.37 |
| chr10:100154960-100154961 | C | T | rs147162823 | 0.31 | 1.05 | 1 | 323 | 6 | 540 | 0.2677 | 84.32 |
| chr10:104196166-104196167 | T | C | rs77128363 | 0.31 | 0.22 | 1 | 323 | 1 | 545 | 1.0000 | 315.00 |
| chr10:105154088-105154089 | A | G | rs7911488 | 32.41 | 36.00 | 105 | 219 | 197 | 349 | 0.3025 | 95.28 |
| chr11:568054-568055 | C | T | - | 0.31 | 0.08 | 1 | 323 | 0 | 546 | 0.3724 | 117.31 |
| chr11:568210-568211 | G | A | rs7395206 | 62.96 | 64.85 | 204 | 120 | 354 | 192 | 0.6089 | 191.82 |
| chr11:43602991-43602992 | A | G | rs201801670 | 0.31 | 0.12 | 1 | 323 | 1 | 545 | 1.0000 | 315.00 |
| chr11:64658725-64658726 | C | T | - | 0.31 | 0.15 | 1 | 323 | 1 | 545 | 1.0000 | 315.00 |
| chr11:64658755-64658756 | G | A | rs111974253 | 0.93 | 0.61 | 3 | 321 | 3 | 543 | 0.6762 | 213.02 |
| chr11:64658835-64658836 | G | A | rs11231898 | 0.31 | 0.10 | 1 | 323 | 1 | 545 | 1.0000 | 315.00 |
| chr11:64659063-64659064 | C | A | - | 0.31 | 0.05 | 1 | 323 | 0 | 546 | 0.3724 | 117.31 |
| chr11:75046262-75046263 | G | A | - | 0.31 | 0.05 | 1 | 323 | 0 | 546 | 0.3724 | 117.31 |
| chr11:121970569-121970570 | G | A | - | 0.31 | 0.11 | 1 | 323 | 1 | 545 | 1.0000 | 315.00 |
| chr11:122022926-122022927 | C | T | rs543412 | 30.75 | 28.34 | 99 | 223 | 155 | 391 | 0.4873 | 153.49 |
| chr11:122022933-122022934 | T | C | - | 0.31 | 0.08 | 1 | 321 | 0 | 546 | 0.3710 | 116.85 |
| chr11:122022963-122022964 | A | C | - | 0.31 | 0.06 | 1 | 321 | 0 | 546 | 0.3710 | 116.85 |
| chr11:122023099-122023100 | T | C | rs11821130 | 0.31 | 0.14 | 1 | 323 | 1 | 545 | 1.0000 | 315.00 |
| chr11:122023186-122023187 | A | G | rs1834306 | 59.01 | 59.33 | 190 | 132 | 324 | 222 | 0.9431 | 297.07 |
| chr12:7073115-7073116 | C | T | - | 0.31 | 0.12 | 1 | 323 | 1 | 545 | 1.0000 | 315.00 |
| chr12:7073121-7073122 | C | T | - | 0.31 | 0.32 | 1 | 323 | 2 | 544 | 1.0000 | 315.00 |
| chr12:7073190-7073191 | A | G | - | 0.31 | 0.54 | 1 | 323 | 3 | 543 | 1.0000 | 315.00 |
| chr12:49048448-49048449 | G | T | - | 0.31 | 0.04 | 1 | 323 | 0 | 546 | 0.3724 | 117.31 |
| chr12:49048505-49048506 | C | T | rs17238458 | 4.63 | 2.78 | 15 | 309 | 15 | 531 | 0.1778 | 56.00 |
| chr12:58218377-58218378 | G | C | - | 0.31 | 0.01 | 1 | 323 | 0 | 546 | 0.3724 | 117.31 |
| chr12:58218381-58218382 | G | A | rs41292017 | 1.54 | 1.64 | 5 | 319 | 9 | 537 | 1.0000 | 315.00 |
| chr12:79812958-79812959 | G | A | - | 0.31 | 0.06 | 1 | 323 | 0 | 546 | 0.3724 | 117.31 |
| chr12:79813069-79813070 | G | A | - | 0.31 | 0.08 | 1 | 323 | 0 | 546 | 0.3724 | 117.31 |
| chr12:81329535-81329536 | A | C | rs2682818 | 91.67 | 85.75 | 297 | 27 | 468 | 78 | 0.0096 | 3.03 |
| chr12:81329671-81329672 | A | G | rs11613504 | 7.10 | 8.17 | 23 | 301 | 45 | 501 | 0.6024 | 189.77 |
| chr12:81329724-81329725 | G | A | rs111921327 | 0.62 | 0.24 | 2 | 322 | 1 | 545 | 0.5592 | 176.16 |
| chr12:81329726-81329727 | G | A | - | 0.31 | 0.18 | 1 | 323 | 1 | 545 | 1.0000 | 315.00 |
| chr12:81329736-81329737 | A | G | rs17732904 | 0.93 | 1.33 | 3 | 321 | 7 | 539 | 0.7518 | 236.81 |
| chr12:81329744-81329745 | C | T | rs144034647 | 0.31 | 0.58 | 1 | 323 | 3 | 543 | 1.0000 | 315.00 |
| chr12:81329755-81329756 | A | G | rs74342046 | 1.85 | 0.08 | 6 | 318 | 0 | 546 | 0.0026 | 0.82 |
| chr12:97885495-97885496 | T | C | rs61943698 | 4.01 | 3.62 | 13 | 311 | 20 | 526 | 0.8549 | 269.29 |
| chr13:50623476-50623477 | T | C | rs41284812 | 0.62 | 2.70 | 2 | 320 | 15 | 531 | 0.0394 | 12.41 |
| chr13:50623485-50623486 | G | A | - | 0.31 | 2.72 | 1 | 321 | 15 | 531 | 0.0081 | 2.54 |
| chr13:50623536-50623537 | C | G | rs41284814 | 0.62 | 2.56 | 2 | 320 | 14 | 532 | 0.0636 | 20.03 |
| chr14:100774202-100774203 | C | T | rs72631832 | 2.16 | 3.50 | 7 | 317 | 19 | 527 | 0.3091 | 97.38 |
| chr14:101318836-101318837 | A | G | rs113399730 | 0.31 | 0.25 | 1 | 323 | 1 | 545 | 1.0000 | 315.00 |
| chr14:101318957-101318958 | C | T | rs144981880 | 0.62 | 0.25 | 2 | 322 | 1 | 545 | 0.5592 | 176.16 |
| chr14:101348365-101348366 | C | T | - | 0.31 | 0.16 | 1 | 323 | 1 | 545 | 1.0000 | 315.00 |
| chr14:101350720-101350721 | T | C | rs139938584 | 0.62 | 0.56 | 2 | 322 | 3 | 543 | 1.0000 | 315.00 |
| chr14:101350813-101350814 | G | T | - | 0.31 | 0.21 | 1 | 323 | 1 | 545 | 1.0000 | 315.00 |
| chr14:101351010-101351011 | T | G | rs186046088 | 0.31 | 0.24 | 1 | 323 | 1 | 545 | 1.0000 | 315.00 |
| chr14:101351026-101351027 | G | A | rs199723831 | 0.31 | 0.31 | 1 | 323 | 2 | 544 | 1.0000 | 315.00 |
| chr14:101351176-101351177 | G | A | rs41286562 | 0.31 | 0.14 | 1 | 323 | 1 | 545 | 1.0000 | 315.00 |
| chr14:101488532-101488533 | G | A | rs8011965 | 0.93 | 0.12 | 3 | 321 | 1 | 545 | 0.1484 | 46.74 |
| chr14:101489702-101489703 | T | C | rs111906529 | 0.93 | 0.60 | 3 | 321 | 3 | 543 | 0.6762 | 213.02 |
| chr14:101492155-101492156 | C | T | rs374951266 | 0.31 | 0.13 | 1 | 323 | 1 | 545 | 1.0000 | 315.00 |
| chr14:101492223-101492224 | T | C | rs7141987 | 46.30 | 41.00 | 150 | 174 | 224 | 322 | 0.1372 | 43.20 |
| chr14:101492547-101492548 | C | T | rs12586258 | 32.10 | 27.02 | 104 | 220 | 148 | 398 | 0.1225 | 38.58 |
| chr14:101493521-101493522 | T | C | - | 0.31 | 0.09 | 1 | 323 | 1 | 545 | 1.0000 | 315.00 |
| chr14:101496122-101496123 | G | A | rs143863696 | 2.16 | 2.00 | 7 | 317 | 11 | 535 | 1.0000 | 315.00 |
| chr14:101496129-101496130 | G | A | rs117518522 | 0.62 | 0.11 | 2 | 322 | 1 | 545 | 0.5592 | 176.16 |
| chr14:101496144-101496145 | A | G | rs9324030 | 45.68 | 39.82 | 148 | 176 | 217 | 329 | 0.0887 | 27.93 |
| chr14:101498223-101498224 | C | T | rs148883152 | 0.31 | 0.13 | 1 | 323 | 1 | 545 | 1.0000 | 315.00 |
| chr14:101498231-101498232 | C | T | rs61992660 | 3.09 | 5.65 | 10 | 314 | 31 | 515 | 0.0977 | 30.79 |
| chr14:101498314-101498315 | G | C | rs118116043 | 0.62 | 2.28 | 2 | 322 | 12 | 534 | 0.0950 | 29.92 |
| chr14:101498356-101498357 | T | C | - | 0.31 | 0.21 | 1 | 323 | 1 | 545 | 1.0000 | 315.00 |
| chr14:101498369-101498370 | A | G | - | 0.31 | 0.08 | 1 | 323 | 0 | 546 | 0.3724 | 117.31 |
| chr14:101498380-101498381 | G | A | - | 0.31 | 0.12 | 1 | 323 | 1 | 545 | 1.0000 | 315.00 |
| chr14:101499988-101499989 | T | A | rs142673707 | 0.31 | 0.57 | 1 | 323 | 3 | 543 | 1.0000 | 315.00 |
| chr14:101500026-101500027 | C | T | rs113479942 | 1.23 | 1.06 | 4 | 320 | 6 | 540 | 1.0000 | 315.00 |
| chr14:101500125-101500126 | G | A | rs367575239 | 0.31 | 0.10 | 1 | 323 | 1 | 545 | 1.0000 | 315.00 |
| chr14:101500132-101500133 | A | G | rs144777170 | 0.31 | 0.08 | 1 | 323 | 0 | 546 | 0.3724 | 117.31 |
| chr14:101500165-101500166 | C | T | rs375457031 | 0.31 | 0.10 | 1 | 323 | 1 | 545 | 1.0000 | 315.00 |
| chr14:101500203-101500204 | C | T | rs372168648 | 0.31 | 0.07 | 1 | 323 | 0 | 546 | 0.3724 | 117.31 |
| chr14:101506009-101506010 | A | G | rs117381856 | 0.62 | 0.56 | 2 | 322 | 3 | 543 | 1.0000 | 315.00 |
| chr14:101506246-101506247 | G | A | - | 0.31 | 0.07 | 1 | 323 | 0 | 546 | 0.3724 | 117.31 |
| chr14:101506276-101506277 | C | T | - | 0.31 | 0.14 | 1 | 323 | 1 | 545 | 1.0000 | 315.00 |
| chr14:101506285-101506286 | A | G | - | 0.62 | 0.28 | 2 | 322 | 2 | 544 | 0.6313 | 198.85 |
| chr14:101506332-101506333 | C | T | rs114035552 | 0.62 | 0.05 | 2 | 322 | 0 | 546 | 0.1384 | 43.60 |
| chr14:101506449-101506450 | G | C | rs376430906 | 0.31 | 0.15 | 1 | 323 | 1 | 545 | 1.0000 | 315.00 |
| chr14:101506532-101506533 | G | T | rs72700572 | 1.23 | 1.22 | 4 | 320 | 7 | 539 | 1.0000 | 315.00 |
| chr14:101506554-101506555 | A | G | rs76847254 | 4.94 | 5.31 | 16 | 308 | 29 | 517 | 0.8751 | 275.67 |
| chr14:101506651-101506652 | C | T | rs147857581 | 0.62 | 0.13 | 2 | 320 | 1 | 545 | 0.5589 | 176.06 |
| chr14:101509224-101509225 | C | G | rs75777456 | 1.23 | 0.68 | 4 | 320 | 4 | 542 | 0.4794 | 151.02 |
| chr14:101509263-101509264 | C | G | rs148213581 | 0.31 | 0.01 | 1 | 323 | 0 | 546 | 0.3724 | 117.31 |
| chr14:101509269-101509270 | C | T | rs112910713 | 1.23 | 1.18 | 4 | 320 | 6 | 540 | 1.0000 | 315.00 |
| chr14:101509273-101509274 | G | A | rs112829040 | 1.23 | 0.78 | 4 | 320 | 4 | 542 | 0.4794 | 151.02 |
| chr14:101509286-101509287 | A | G | rs7147329 | 7.72 | 11.27 | 25 | 299 | 62 | 484 | 0.1013 | 31.92 |
| chr14:101509448-101509449 | G | A | rs189513510 | 0.62 | 0.30 | 2 | 322 | 2 | 544 | 0.6313 | 198.85 |
| chr14:101510399-101510400 | G | T | rs142240732 | 0.31 | 1.24 | 1 | 323 | 7 | 539 | 0.2700 | 85.05 |
| chr14:101510612-101510613 | G | A | rs11844707 | 0.31 | 0.08 | 1 | 323 | 0 | 546 | 0.3724 | 117.31 |
| chr14:101513794-101513795 | C | T | rs72700576 | 4.01 | 2.95 | 13 | 311 | 16 | 530 | 0.4362 | 137.41 |
| chr14:101514370-101514371 | T | C | rs10132916 | 28.09 | 27.21 | 91 | 233 | 149 | 397 | 0.8142 | 256.47 |
| chr14:101518759-101518760 | G | A | rs1951032 | 22.22 | 19.27 | 72 | 252 | 105 | 441 | 0.2969 | 93.51 |
| chr14:101518772-101518773 | G | A | - | 0.31 | 0.11 | 1 | 323 | 1 | 545 | 1.0000 | 315.00 |
| chr14:101520526-101520527 | A | C | rs28752084 | 23.15 | 21.11 | 75 | 249 | 115 | 431 | 0.4975 | 156.70 |
| chr14:101520672-101520673 | C | T | - | 0.31 | 0.10 | 1 | 323 | 1 | 545 | 1.0000 | 315.00 |
| chr14:101520828-101520829 | C | T | rs77282763 | 0.62 | 0.52 | 2 | 322 | 3 | 543 | 1.0000 | 315.00 |
| chr14:101521153-101521154 | G | A | rs377391508 | 0.31 | 0.14 | 1 | 323 | 1 | 545 | 1.0000 | 315.00 |
| chr14:101522555-101522556 | T | C | rs56103835 | 22.53 | 21.30 | 73 | 251 | 116 | 430 | 0.6712 | 211.44 |
| chr14:101522588-101522589 | C | T | rs75330474 | 0.62 | 0.59 | 2 | 322 | 3 | 543 | 1.0000 | 315.00 |
| chr14:101526180-101526181 | G | A | rs41286572 | 4.63 | 6.36 | 15 | 309 | 35 | 511 | 0.2958 | 93.17 |
| chr14:101527032-101527033 | C | T | rs78425563 | 0.31 | 0.54 | 1 | 323 | 3 | 543 | 1.0000 | 315.00 |
| chr14:101527117-101527118 | C | T | rs117924003 | 3.70 | 4.43 | 12 | 312 | 24 | 522 | 0.7258 | 228.63 |
| chr14:101528523-101528524 | C | T | rs117311466 | 0.93 | 1.36 | 3 | 321 | 7 | 539 | 0.7518 | 236.81 |
| chr14:101528539-101528540 | C | T | - | 0.62 | 0.56 | 2 | 322 | 3 | 543 | 1.0000 | 315.00 |
| chr14:101530728-101530729 | G | A | - | 0.31 | 0.33 | 1 | 323 | 2 | 544 | 1.0000 | 315.00 |
| chr14:101530932-101530933 | C | T | rs143440903 | 0.62 | 0.40 | 2 | 322 | 2 | 544 | 0.6313 | 198.85 |
| chr14:101531567-101531568 | G | C | rs150699693 | 0.62 | 1.15 | 2 | 322 | 6 | 540 | 0.7171 | 225.88 |
| chr14:101531732-101531733 | C | T | rs61992670 | 3.40 | 6.19 | 11 | 313 | 34 | 512 | 0.0812 | 25.58 |
| chr14:101531733-101531734 | G | A | rs201007039 | 0.31 | 0.72 | 1 | 323 | 4 | 542 | 0.6562 | 206.69 |
| chr14:101531853-101531854 | A | G | rs61992671 | 43.21 | 45.91 | 140 | 184 | 251 | 295 | 0.4389 | 138.24 |
| chr14:101532216-101532217 | G | A | rs10144831 | 4.01 | 6.54 | 13 | 311 | 36 | 510 | 0.1287 | 40.53 |
| chr14:101532903-101532904 | C | T | rs372816450 | 0.62 | 0.17 | 2 | 322 | 1 | 545 | 0.5592 | 176.16 |
| chr14:101533092-101533093 | C | T | rs58834075 | 2.78 | 3.53 | 9 | 315 | 19 | 527 | 0.6924 | 218.12 |
| chr14:101533126-101533127 | C | T | rs371731233 | 0.62 | 0.10 | 2 | 322 | 1 | 545 | 0.5592 | 176.16 |
| chr15:55665088-55665089 | T | C | rs8041885 | 8.33 | 10.55 | 27 | 297 | 58 | 488 | 0.2899 | 91.33 |
| chr15:55665097-55665098 | T | C | rs115010895 | 0.31 | 0.30 | 1 | 323 | 2 | 544 | 1.0000 | 315.00 |
| chr15:55665199-55665200 | T | C | rs200771662 | 0.31 | 0.04 | 1 | 323 | 0 | 546 | 0.3724 | 117.31 |
| chr15:55665312-55665313 | C | A | rs8041044 | 8.33 | 10.51 | 27 | 297 | 57 | 489 | 0.3433 | 108.14 |
| chr15:70371723-70371724 | G | A | rs369490351 | 0.31 | 0.09 | 1 | 323 | 1 | 545 | 1.0000 | 315.00 |
| chr15:70371776-70371777 | C | G | rs78212770 | 0.93 | 1.21 | 3 | 321 | 7 | 539 | 0.7518 | 236.81 |
| chr15:70371866-70371867 | C | T | rs79128009 | 0.93 | 1.65 | 3 | 321 | 9 | 537 | 0.5505 | 173.40 |
| chr15:70371873-70371874 | G | C | rs75165322 | 0.93 | 1.74 | 3 | 321 | 9 | 537 | 0.5505 | 173.40 |
| chr15:89151215-89151216 | T | C | - | 0.31 | 0.24 | 1 | 323 | 1 | 545 | 1.0000 | 315.00 |
| chr15:89154936-89154937 | T | A | rs111791075 | 0.62 | 0.55 | 2 | 322 | 3 | 543 | 1.0000 | 315.00 |
| chr15:89154937-89154938 | G | A | - | 0.31 | 0.06 | 1 | 323 | 0 | 546 | 0.3724 | 117.31 |
| chr15:89154953-89154954 | A | G | rs28667006 | 0.62 | 0.95 | 2 | 322 | 5 | 541 | 1.0000 | 315.00 |
| chr15:89154965-89154966 | G | A | rs7170666 | 3.40 | 3.52 | 11 | 313 | 19 | 527 | 1.0000 | 315.00 |
| chr15:89154999-89155000 | C | T | rs111470135 | 0.31 | 0.10 | 1 | 323 | 1 | 545 | 1.0000 | 315.00 |
| chr15:89155013-89155014 | C | T | rs41276928 | 35.49 | 31.49 | 115 | 209 | 172 | 374 | 0.2334 | 73.52 |
| chr15:89155072-89155073 | C | T | rs41276930 | 0.31 | 0.29 | 1 | 323 | 2 | 544 | 1.0000 | 315.00 |
| chr16:2321820-2321821 | G | A | rs369201720 | 0.31 | 0.25 | 1 | 323 | 1 | 545 | 1.0000 | 315.00 |
| chr16:14397923-14397924 | T | C | rs112887255 | 0.93 | 1.74 | 3 | 321 | 10 | 536 | 0.3912 | 123.24 |
| chr16:56892592-56892593 | G | A | rs76987351 | 1.54 | 2.21 | 5 | 319 | 12 | 534 | 0.6169 | 194.33 |
| chr16:67236092-67236093 | C | T | rs368446112 | 0.31 | 0.09 | 1 | 323 | 1 | 545 | 1.0000 | 315.00 |
| chr16:67236101-67236102 | C | T | rs200402288 | 0.31 | 0.12 | 1 | 323 | 1 | 545 | 1.0000 | 315.00 |
| chr16:67236108-67236109 | A | T | rs192034781 | 0.93 | 0.82 | 3 | 321 | 4 | 542 | 0.7157 | 225.43 |
| chr16:67236291-67236292 | C | G | rs188892061 | 0.62 | 0.53 | 2 | 322 | 3 | 543 | 1.0000 | 315.00 |
| chr17:6920887-6920888 | C | T | rs41283391 | 7.41 | 5.95 | 24 | 300 | 33 | 513 | 0.4791 | 150.91 |
| chr17:17717270-17717271 | C | T | rs115111429 | 0.31 | 0.16 | 1 | 323 | 1 | 545 | 1.0000 | 315.00 |
| chr17:19247820-19247821 | C | T | - | 0.31 | 0.13 | 1 | 323 | 1 | 545 | 1.0000 | 315.00 |
| chr17:27188677-27188678 | G | A | rs139953196 | 0.31 | 0.61 | 1 | 321 | 3 | 543 | 1.0000 | 315.00 |
| chr17:28444046-28444047 | A | T | - | 0.31 | 0.01 | 1 | 323 | 0 | 546 | 0.3724 | 117.31 |
| chr17:28444253-28444254 | T | A | rs8067576 | 43.83 | 43.47 | 142 | 182 | 237 | 309 | 0.9436 | 297.25 |
| chr17:29902588-29902589 | C | T | rs138149844 | 0.31 | 0.35 | 1 | 323 | 2 | 544 | 1.0000 | 315.00 |
| chr17:46114619-46114620 | C | T | rs12940701 | 29.32 | 31.77 | 95 | 229 | 173 | 373 | 0.4946 | 155.79 |
| chr17:46114662-46114663 | C | A | - | 0.31 | 0.04 | 1 | 323 | 0 | 546 | 0.3724 | 117.31 |
| chr17:46114691-46114692 | C | G | rs41280120 | 17.90 | 19.38 | 58 | 266 | 106 | 440 | 0.5922 | 186.54 |
| chr17:46657177-46657178 | A | T | rs3809783 | 0.62 | 3.17 | 2 | 322 | 17 | 529 | 0.0151 | 4.75 |
| chr17:46657179-46657180 | T | A | - | 0.31 | 1.04 | 1 | 323 | 6 | 540 | 0.2677 | 84.32 |
| chr17:56408585-56408586 | C | T | - | 0.31 | 0.10 | 1 | 323 | 1 | 545 | 1.0000 | 315.00 |
| chr18:19405587-19405588 | T | G | - | 0.31 | 0.05 | 1 | 323 | 0 | 546 | 0.3724 | 117.31 |
| chr18:19408870-19408871 | C | T | rs369294491 | 0.31 | 0.09 | 1 | 323 | 0 | 546 | 0.3724 | 117.31 |
| chr18:19408890-19408891 | A | G | rs78641532 | 10.80 | 10.69 | 35 | 289 | 58 | 488 | 1.0000 | 315.00 |
| chr18:19408949-19408950 | C | T | rs9989532 | 99.38 | 97.87 | 322 | 2 | 534 | 12 | 0.0950 | 29.92 |
| chr18:33484791-33484792 | G | A | rs41274312 | 1.54 | 0.97 | 5 | 319 | 5 | 541 | 0.5131 | 161.64 |
| chr18:56118103-56118104 | C | T | - | 0.31 | 0.39 | 1 | 323 | 2 | 544 | 1.0000 | 315.00 |
| chr18:56118220-56118221 | A | G | - | 0.31 | 0.08 | 1 | 323 | 0 | 546 | 0.3724 | 117.31 |
| chr18:56118277-56118278 | A | T | rs111316406 | 0.31 | 0.02 | 1 | 323 | 0 | 546 | 0.3724 | 117.31 |
| chr18:56118357-56118358 | C | T | rs41292412 | 1.23 | 1.59 | 4 | 320 | 9 | 537 | 0.7764 | 244.58 |
| chr19:10928178-10928179 | G | A | rs373701104 | 0.31 | 0.56 | 1 | 323 | 3 | 543 | 1.0000 | 315.00 |
| chr19:13947291-13947292 | T | C | rs895819 | 34.88 | 35.65 | 113 | 211 | 195 | 351 | 0.8261 | 260.23 |
| chr19:13947295-13947296 | G | A | rs11671784 | 1.85 | 1.53 | 6 | 318 | 8 | 538 | 0.7817 | 246.23 |
| chr19:40788441-40788442 | T | G | rs73933241 | 8.33 | 7.53 | 27 | 297 | 41 | 505 | 0.6958 | 219.18 |
| chr19:40788640-40788641 | T | C | rs11880261 | 73.46 | 70.24 | 238 | 86 | 384 | 162 | 0.3515 | 110.72 |
| chr19:50004165-50004166 | C | T | rs41275778 | 0.62 | 0.80 | 2 | 322 | 4 | 542 | 1.0000 | 315.00 |
| chr19:52196408-52196409 | G | A | rs41275794 | 2.80 | 3.25 | 9 | 313 | 18 | 528 | 0.8401 | 264.64 |
| chr19:52196487-52196488 | G | A | rs10404453 | 0.31 | 0.28 | 1 | 319 | 1 | 489 | 1.0000 | 315.00 |
| chr19:54240053-54240054 | G | A | rs201913824 | 0.62 | 0.41 | 2 | 322 | 2 | 544 | 0.6313 | 198.85 |
| chr19:54240087-54240088 | G | A | rs77959394 | 5.25 | 6.23 | 17 | 307 | 34 | 512 | 0.6548 | 206.25 |
| chr19:54240218-54240219 | G | A | rs141076796 | 1.85 | 2.45 | 6 | 318 | 13 | 533 | 0.8110 | 255.48 |
| chr19:54485424-54485425 | C | T | - | 0.31 | 0.11 | 1 | 321 | 1 | 531 | 1.0000 | 315.00 |
| chr19:54485711-54485712 | G | C | - | 0.31 | 0.00 | 1 | 321 | 0 | 518 | 0.3833 | 120.75 |
| chr20:57392685-57392686 | G | A | rs117258475 | 2.47 | 0.20 | 8 | 316 | 1 | 545 | 0.0021 | 0.67 |
| chr20:61151635-61151636 | C | T | rs190492357 | 0.62 | 0.05 | 2 | 322 | 0 | 546 | 0.1384 | 43.60 |
| chr20:61151713-61151714 | G | A | rs74510555 | 0.31 | 0.72 | 1 | 321 | 4 | 542 | 0.6565 | 206.79 |
| chr20:61162084-61162085 | G | A | - | 0.31 | 0.09 | 1 | 323 | 0 | 546 | 0.3724 | 117.31 |
| chr20:61162099-61162100 | G | A | rs13040413 | 23.46 | 21.95 | 76 | 248 | 120 | 426 | 0.6155 | 193.89 |
| chr20:61162218-61162219 | C | T | rs370228698 | 0.31 | 0.11 | 1 | 323 | 1 | 545 | 1.0000 | 315.00 |
| chr20:61162266-61162267 | T | C | rs6062251 | 60.19 | 55.08 | 195 | 129 | 301 | 245 | 0.1568 | 49.38 |
| chr20:61162288-61162289 | G | A | rs149629841 | 0.31 | 0.36 | 1 | 323 | 2 | 544 | 1.0000 | 315.00 |
| chr21:17912308-17912309 | G | A | rs78999419 | 0.31 | 0.10 | 1 | 323 | 1 | 545 | 1.0000 | 315.00 |
| chr21:17962381-17962382 | T | A | rs2823897 | 2.78 | 1.39 | 9 | 315 | 8 | 538 | 0.2076 | 65.39 |
| chr22:20236631-20236632 | T | C | rs9618761 | 0.93 | 0.18 | 3 | 321 | 1 | 545 | 0.1484 | 46.74 |
| chr22:20236780-20236781 | G | A | rs370242881 | 0.31 | 0.18 | 1 | 323 | 1 | 545 | 1.0000 | 315.00 |
| chr22:42296906-42296907 | T | G | - | 0.31 | 0.94 | 1 | 323 | 5 | 541 | 0.4203 | 132.38 |
| chrX:49767831-49767832 | A | G | rs456615 | 100.00 | 99.88 | 324 | 0 | 545 | 1 | 1.0000 | 315.00 |
| chrX:49767834-49767835 | A | G | rs456617 | 100.00 | 99.92 | 324 | 0 | 546 | 0 | 1.0000 | 315.00 |
| chrX:49767903-49767904 | A | G | rs149259 | 100.00 | 99.88 | 324 | 0 | 545 | 1 | 1.0000 | 315.00 |
| chrX:49773306-49773307 | T | G | rs59038115 | 0.31 | 0.02 | 1 | 323 | 0 | 546 | 0.3724 | 117.31 |
| chrX:49773804-49773805 | A | C | rs149596 | 100.00 | 99.92 | 324 | 0 | 546 | 0 | 1.0000 | 315.00 |
| chrX:49774446-49774447 | C | T | rs149202543 | 6.48 | 4.47 | 21 | 303 | 24 | 522 | 0.2055 | 64.75 |
| chrX:73438223-73438224 | C | T | rs372055341 | 0.62 | 1.00 | 2 | 322 | 5 | 541 | 1.0000 | 315.00 |
| chrX:133303802-133303803 | A | G | rs201499941 | 0.31 | 0.08 | 1 | 323 | 0 | 546 | 0.3724 | 117.31 |
| chrX:133680633-133680634 | G | A | rs373943888 | 0.31 | 0.93 | 1 | 323 | 5 | 541 | 0.4203 | 132.38 |
| chrX:139006270-139006271 | C | G | rs139318512 | 0.31 | 0.62 | 1 | 323 | 3 | 543 | 1.0000 | 315.00 |
| chrX:146341310-146341311 | C | A | rs78095394 | 3.40 | 2.41 | 11 | 313 | 13 | 533 | 0.3969 | 125.03 |
| chrX:146342027-146342028 | C | T | rs187006550 | 0.62 | 1.43 | 2 | 320 | 8 | 538 | 0.3377 | 106.38 |
| chrX:146342054-146342055 | G | T | - | 0.31 | 0.25 | 1 | 321 | 1 | 545 | 1.0000 | 315.00 |
| chrX:146342232-146342233 | T | C | rs73243481 | 0.93 | 1.27 | 3 | 319 | 7 | 539 | 0.7522 | 236.94 |
| chrX:146342250-146342251 | C | T | rs7885181 | 1.55 | 2.50 | 5 | 317 | 14 | 532 | 0.4720 | 148.68 |


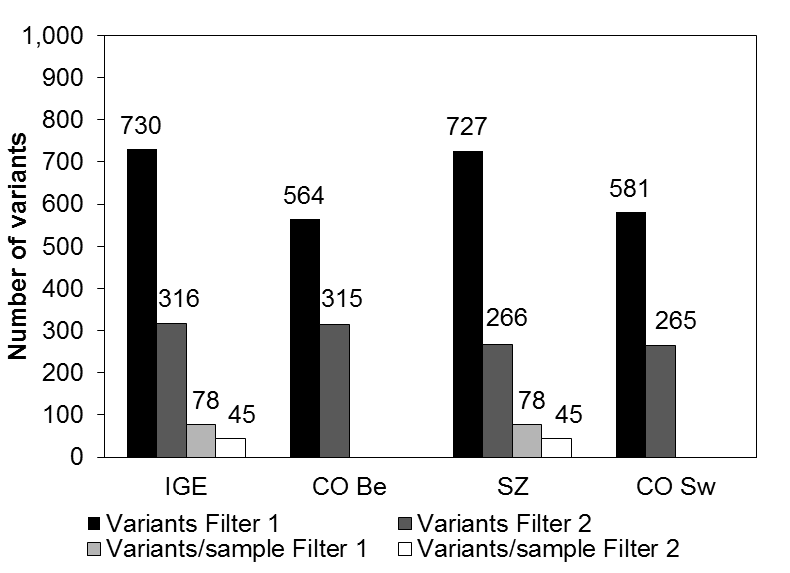
**Fig A. Total number of variants identified in the different groups, after filter step 1 and filter step 2 and average number of variants per sample for patient groups.** IGE: patients with idiopathic generalized epilepsy; CO Be: Belgian/Dutch control samples. SZ: schizophrenia patients; CO Sw: Swedish control samples.

**Table C. Genetic variants with different frequency in the schizophrenia patients versus control individuals (p < 0.05).**

| **Genomic coordinates** | **Ref>Alt** | **SNP ID** | **miRNA** | **Location** | **P** | **Padj** | **Freq (SZ)** | **Freq (Co Sw)** | **1000g low (EUR)** |
| --- | --- | --- | --- | --- | --- | --- | --- | --- | --- |
| chr1:94312565-94312566 | G>T | rs2391318 | *MIR760* | Flank | 0.0100 | 2.64 | 100 | 98.49 | - |
| chr1:94312566-94312567 | T>C | rs2391319 | *MIR760* | Flank | 0.0155 | 4.10 | 100 | 98.66 | - |
| chr7:1062795-1062796 | T>C | rs113664658 | *MIR339* | Flank | 0.0499 | 13.23 | 1.34 | 0.42 | 1.00 |
| chr9:73424783-73424784 | C>T | rs7861254 | *MIR204* | Flank | 0.0002 | 0.04 | 16.39 | 25.34 | 22.00 |
| chr12:49048505-49048506 | C>T | rs17238458 | *MIR1291* | Flank | 0.0461 | 12.22 | 2.7 | 5.08 | 2.00 |
| chr19:10928178-10928179 | G>A | rs373701104 | *MIR199A1* | Flank | 0.0240 | 6.37 | 1.35 | 0.34 | - |
| chrX:49774446-49774447 | C>T | rs149202543 | *MIR501* | Flank | 0.0011 | 0.29 | 5.68 | 2.35 | 2.00 |
| chrX:146342027-146342028 | C>T | rs187006550 | *MIR509-1* | Flank | 0.0078 | 2.05 | 1.62 | 0.33 | 1.00 |
| chrX:146342250-146342251 | C>T | rs7885181 | *MIR509-1* | Flank | 0.0242 | 6.42 | 1.62 | 0.48 | 1.00 |
| chrX:151562021-151562022 | T>A | rs184094665 | *MIR767* | Flank | 0.0014 | 0.37 | 1.62 | 0.21 | 1.00 |

Location: relative to the miRNA gene. P: Fisher’s exact p-value (unadjusted). Padj: Bonferroni adjusted Fisher’s exact p-value. Freq: frequency (%) of alternative allele. Co Sw: Swedish controls. 1000g low (EUR): alternative allele frequency in 1000Genomes, European population.

**Table D. Genetic variants different frequency in the IGE patients versus control individuals (p < 0.05).**

| **Genomic coordinates** | **Ref>Alt** | **SNP ID** | **miRNA** | **Location** | **P** | **Padj** | **Freq (IGE)** | **Freq (CO Be)** | **1000g low (EUR)** |
| --- | --- | --- | --- | --- | --- | --- | --- | --- | --- |
| chr1:94312565-94312566 | G>T | rs2391318 | *MIR760* | Flank | 0.0164 | 5.16 | 100 | 98.18 | - |
| chr1:94312566-94312567 | T>C | rs2391319 | *MIR760* | Flank | 0.0306 | 9.64 | 100 | 98.39 | - |
| chr12:81329535-81329536 | A>C | rs2682818 | *MIR618* | Precursor | 0.0096 | 3.03 | 91.67 | 85.75 | 86.00 |
| chr12:81329755-81329756 | A>G | rs74342046 | *MIR618* | Flank | 0.0026 | 0.82 | 1.85 | 0.08 | 1.00 |
| chr13:50623476-50623477 | T>C | rs41284812 | *MIR15A* | Flank | 0.0394 | 12.41 | 0.62 | 2.7 | 2.00 |
| chr13:50623485-50623486 | G>A | - | *MIR15A* | Flank | 0.0081 | 2.54 | 0.31 | 2.72 | - |
| chr17:46657177-46657178 | A>T | rs3809783 | *MIR10A* | Flank | 0.0151 | 4.75 | 0.62 | 3.17 | 1.00 |
| chr20:57392685-57392686 | G>A | rs117258475 | *MIR296* | Mature | 0.0021 | 0.67 | 2.47 | 0.2 | 1.00 |

# Co Be: Belgian/Dutch controls.

# Expression analysis


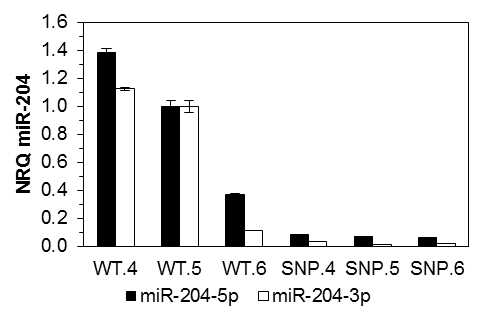

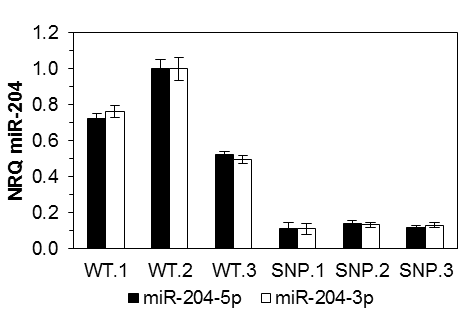


**a b**


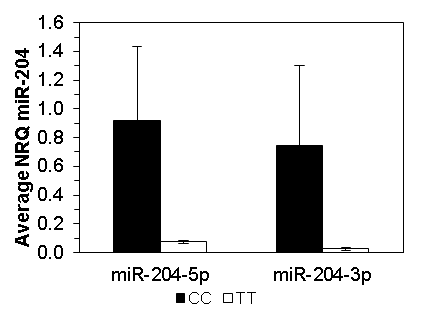
**Fig B. Normalized relative quantities (NRQ) of miR-204 in *MIR204* cells replicate 1 (panel (a), clones 1-3) and replicate 2 (panel (b), clones 4-6).** Error bars represent the error on the NRQ ratio of miRNA/EGFP, calculated by error propagation of the standard deviation of the NRQ.

**Fig C. Average NRQs of miR-204 for replicate 2 (*MIR204* cells, clones 4-6).** Error bars represent standard deviation of biological triplicates. The fold changes between TT (MIR204SNP) and CC (MIR204WT) cells are 0.080 and 0.034, for miR-204-5p and miR-204-3p respectively. Clone WT.6 has aberrant expression and results in a high standard deviation.

## Transcriptome analysis

**SAM analysis**


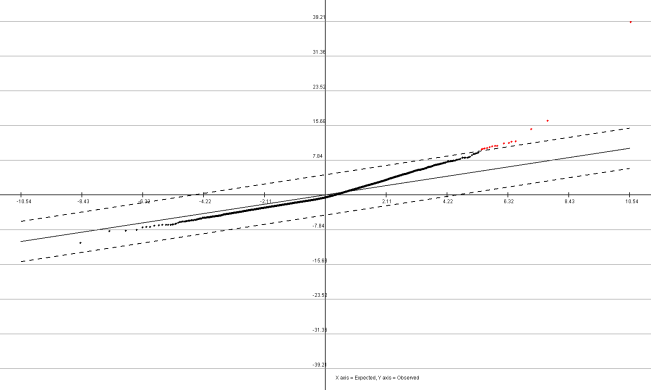

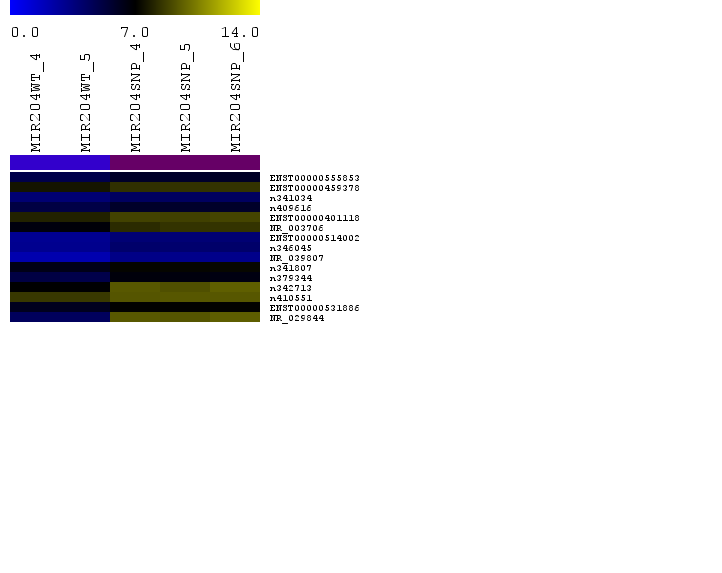


**a b**

**Fig D. SAM comparing MIR204WT with MIR204SNP.** (a) SAM graph; (b) expression image. 10 unique permutations were used. Delta: 4.529. Upper cutoff: 10.485417, lower cutoff: -infinity, FDR (median, 90th percentile): 0.00000%.


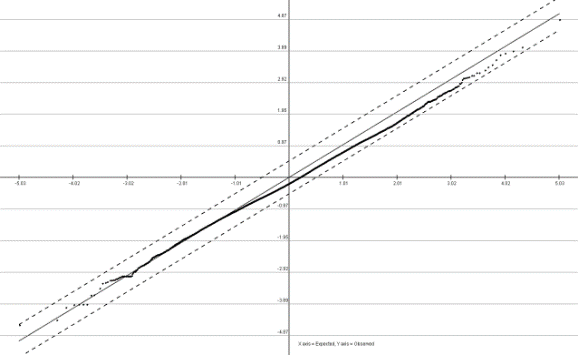


**Fig E. SAM graph for comparing MIR618WT with MIR618SNP.** 20 unique permutations were performed. Delta: 0.500. No significant genes were identified. Upper cutoff: infinity, lower cutoff: -infinity, FDR (median, 90th percentile): NA.

# RNAhybrid predictions

**Table E. RNAhybrid target prediction results for miR-204-5p and miR-204-3p.**

|  |  |  | **Target sites** | | | | |
| --- | --- | --- | --- | --- | --- | --- | --- |
| **Transcript** | **Techn** | **Size** | **5p** | **3p** | **Total** | **Per 500 bp** | **Expr** |
| pri-miR-296 | M,S | 480 | 1 | 0 | 1 | 1.0 | - |
| pri-miR-138-1 | S | 499 | 0 | 2 | 2 | 2.0 | - |
| pri-miR-744 | S | 498 | 0 | 0 | 0 | 0.0 | - |
| pri-let-7i | S | 484 | 2 | 1 | 3 | 3.1 | - |
| pri-miR-4659b | M | 473 | 0 | 0 | 0 | 0.0 | - |
| pri-miR-7641-1, pri-miR-7641-2 | S | 914 | 0 | 0 | 0 | 0.0 | + |
| pri-miR-7-1, pri-miR-7-2, pri-miR-7-3 | S | 1530 | 2 | 2 | 4 | 1.3 | + |
| ENST00000363524 (NR_003706) | M | 131 | 0 | 0 | 0 | 0.0 | - |
| ENST00000514002 | M | 439 | 0 | 0 | 0 | 0.0 | - |
| ENST00000555853 | M | 300 | 0 | 1 | 1 | 1.7 | - |
| ENST00000531886 | M | 667 | 1 | 0 | 1 | 0.7 | - |
| NONHSAT021478 (n341034) | M | 1094 | 2 | 1 | 3 | 1.4 | - |
| NONHSAT123639 (n409616) | M | 460 | 0 | 0 | 0 | 0.0 | - |
| NONHSAT135470 (n341807) | M | 1234 | 0 | 0 | 0 | 0.0 | - |
| NONHSAT048382 (n346045) | M | 2496 | 0 | 0 | 0 | 0.0 | - |
| NONHSAT125770 (n379344) | M | 6111 | 10 | 5 | 15 | 1.2 | - |
| NONHSAT054325 (n342713) | M | 267 | 0 | 0 | 0 | 0.0 | - |
| NR_037718.1 (n410551) | M | 1936 | 3 | 0 | 3 | 0.8 | - |

# Transcript: transcripts with significantly different expression between MIR204WT and SNP cells from microarray analysis and small RNA sequencing experiments, excluding two retired transcripts. For some of the differentially expressed mature miRNAs, the miRNA can be transcribed from several loci. The primary miRNA transcript was defined as the hairpin and 200 bp of flanking sequences. For transcripts of the microarray analysis, accession number as given by annotation of the array is given between brackets. Techn: technology in which transcript was found to be significantly altered: M: micro-array, S: small RNA sequencing. Size: total transcript size (in bp), summed over different loci. Target sites: RNAhybrid results. 5p/3p: number of target sites for miR-204-5p or -3p predicted in the transcript. Total: sum of 5p and 3p target sites. Per 500 bp: total target sites normalized for transcript length and multiplied by 500 bp to enable comparisons between transcripts with different lengths. Expr: expression correlation between miR-204 and transcript. “-” when miR-204 and transcript correlation is negative, “+” when it is positive.

# Small RNA sequencing


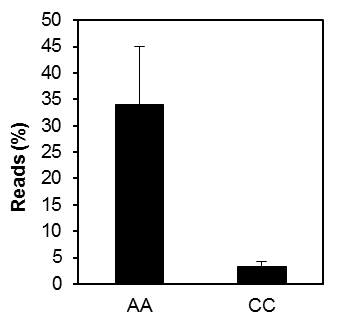

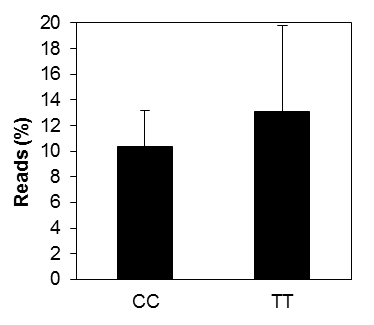

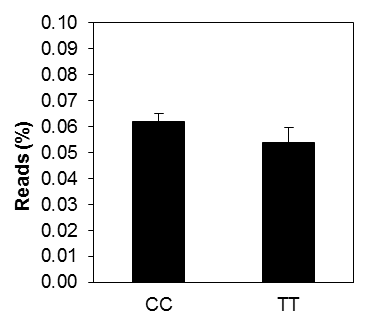


**a b c**

**Fig F. Frequencies of 5’ change isomiRs of miR-618 (panel a) and miR-204 (panels b-c).** (a) Average frequency of 5’ change isomiRs of miR-618 in *MIR618* cells with different genotypes (AA: MIR618WT; CC: MIR618SNP). (b-c) Average frequency of 5’ change isomiRs of miR-204-5p (b) and miR-204-3p (c) in *MIR204* cells with different genotypes (CC: MIR204WT, TT: MIR204SNP). Error bars represent standard deviation of biological replicates.

**References**

1. Landgraf P, Rusu M, Sheridan R, Sewer A, Iovino N, Aravin A, et al. A mammalian microRNA expression atlas based on small RNA library sequencing. Cell. 2007;129: 1401–1414. doi:10.1016/j.cell.2007.04.040

2. Forero DA, van der Ven K, Callaerts P, Del-Favero J. miRNA genes and the brain: implications for psychiatric disorders. Hum Mutat. 2010;31: 1195–1204. doi:10.1002/humu.21344

3. Shin C, Nam J-W, Farh KK-H, Chiang HR, Shkumatava A, Bartel DP. Expanding the microRNA targeting code: functional sites with centered pairing. Mol Cell. 2010;38: 789–802. doi:10.1016/j.molcel.2010.06.005

4. Faghihi MA, Zhang M, Huang J, Modarresi F, Van der Brug MP, Nalls MA, et al. Evidence for natural antisense transcript-mediated inhibition of microRNA function. Genome Biol. 2010;11: R56. doi:10.1186/gb-2010-11-5-r56

5. Hu HY, Guo S, Xi J, Yan Z, Fu N, Zhang X, et al. MicroRNA expression and regulation in human, chimpanzee, and macaque brains. PLoS Genet. 2011;7: e1002327. doi:10.1371/journal.pgen.1002327

6. Goossens D, Moens LN, Nelis E, Lenaerts A-S, Glassee W, Kalbe A, et al. Simultaneous mutation and copy number variation (CNV) detection by multiplex PCR-based GS-FLX sequencing. Hum Mutat. 2009;30: 472–476. doi:10.1002/humu.20873

7. Moens LN, De Rijk P, Reumers J, Van den Bossche MJA, Glassee W, De Zutter S, et al. Sequencing of DISC1 pathway genes reveals increased burden of rare missense variants in schizophrenia patients from a northern Swedish population. PloS One. 2011;6: e23450. doi:10.1371/journal.pone.0023450

8. Li H, Durbin R. Fast and accurate long-read alignment with Burrows-Wheeler transform. Bioinforma Oxf Engl. 2010;26: 589–595. doi:10.1093/bioinformatics/btp698

9. DePristo MA, Banks E, Poplin R, Garimella KV, Maguire JR, Hartl C, et al. A framework for variation discovery and genotyping using next-generation DNA sequencing data. Nat Genet. 2011;43: 491–498. doi:10.1038/ng.806

10. Li H, Handsaker B, Wysoker A, Fennell T, Ruan J, Homer N, et al. The Sequence Alignment/Map format and SAMtools. Bioinforma Oxf Engl. 2009;25: 2078–2079. doi:10.1093/bioinformatics/btp352

11. Kozomara A, Griffiths-Jones S. miRBase: integrating microRNA annotation and deep-sequencing data. Nucleic Acids Res. 2011;39: D152–157. doi:10.1093/nar/gkq1027

12. Reumers J, De Rijk P, Zhao H, Liekens A, Smeets D, Cleary J, et al. Optimized filtering reduces the error rate in detecting genomic variants by short-read sequencing. Nat Biotechnol. 2012;30: 61–68. doi:10.1038/nbt.2053

13. Cammaerts S, Strazisar M, Dierckx J, Del Favero J, De Rijk P. miRVaS: a tool to predict the impact of genetic variants on miRNAs. Nucleic Acids Res. 2015; doi:10.1093/nar/gkv921
