# Supplementary material for: Schizophrenia-Associated MIR204 Regulates Noncoding RNAs and Affects Neurotransmitter and Ion Channel Gene Sets
Source: PLoS One. 2015 Dec 29;10(12):e0144428. doi: 10.1371/journal.pone.0144428 (PMC4695081; doi:10.1371/journal.pone.0144428)
Supplement: S2 File — This file contains additional details of the methods. (DOCX) [file pone.0144428.s002.docx]

# S2. Supplementary Methods

### **Cloning and cell lines**

miRNA gene inserts were amplified from genomic DNA as follows. In each 50µl PCR reaction, 0.6µl of 2.5U/µl Platinum Pfx DNA Polymerase, 5µl of 10x Pfx Amplification buffer, 1µl of 50mM MgSO_4_, 0.6µl of 25mM dNTP mix (previous products all Life technologies, CA, USA), 1.5µl of 10µM forward primer (IDT, Integrated DNA Technologies, CA, USA), 1.5µl of 10µM reverse primer (IDT), 2µl of 10ng/µl genomic DNA and 37.8µl H_2_O were combined. PCR condition: denaturation for 2 min at 94 °C, followed by 38 cycles of (15 sec at 94°C, 30 sec at 60.4°C and 1 min at 68 °C) and final elongation of 6 min at 68°C on a Primus 96 advanced Gradient thermocycler (Peqlab, VWR, PA, USA). PCR products were bead-purified using Agencourt AMPure XP beads (Beckman Coulter, CA, USA) in a sample:beads ratio of 1:0.8 prior to recombination.

Sequences of primers used for cloning:

| **Primer** | **Sequence (5'-3')** |
| --- | --- |
| attB1_MIR204F | GGGGACAAGTTTGTACAAAAAAGCAGGCTCCTCCTGATCATTTACCCACAG |
| attB2_MIR204R | GGGGACCACTTTGTACAAGAAAGCTGGGTCCTTCCTAATTCCAGAGCTGC |
| attB1_MIR618F | GGGGACAAGTTTGTACAAAAAAGCAGGCTTCCCATTACAAAATCACAGTCGT |
| attB2_MIR618R | GGGGACCACTTTGTACAAGAAAGCTGGGTTCAGAGTTGCAGCTAGGACC |

Stable SH-SY5Y cell lines were named as follows:

MIR204WT: cells overexpressing wild type construct of *MIR204*

MIR204SNP: cells overexpressing variant construct of *MIR204*

MIR618WT: cells overexpressing wild type construct of *MIR618*

MIR618SNP: cells overexpressing variant construct of *MIR618*

Clones 1-3: first triplicate of cell clones from parental stable cell line

Clones 4-6: second triplicate of cell clones from parental stable cell line

### **RNA isolation**

RNA was extracted with mirVana miRNA Isolation Kit (Ambion, Life Technologies) from stable cells according to the manufacturer’s protocol. RNA was subsequently DNase treated with the TURBO DNA-free Kit (Ambion, Life Technologies) according to the supplier’s protocol. RNA concentration was measured on Nanodrop (Thermo Scientific, DE, USA). Where needed, RNA was purified and concentrated by RNA precipitation. Integrity of samples sent for microarray analysis and small RNA sequencing was assessed on Agilent 2100 Bioanalyzer with Agilent RNA 6000 Nano kits (Agilent Technologies, CA, USA).

### **miRNA and EGFP expression analysis**

Reverse transcription of miRNAs was performed according to the manufacturer’s protocol for creating custom RT pools with minor modifications (primerpool concentration: 1:500, no preamplification) using RT primers of following TaqMan microRNA assays (Life Technologies): hsa-miR-204 (ID 000508), mmu-miR-204* (ID 463101_mat), hsa-miR-618 (ID 001593), RNU24 (ID 001001), hsa-miR-16 (ID 000391), hsa-miR-199a (ID 000498), hsa-miR-199a-3p (ID 002304), hsa-miR-423 (ID 000576), hsa-miR-374 (ID 000563). QPCR analysis was performed using the Taqman probes of the hsa-miR-204, mmu-miR-204*, hsa-miR-618, RNU24 and hsa-miR-16 assays. For EGFP expression analysis, reverse transcription was performed using SuperScript III First-Strand Synthesis System (Invitrogen, Life Technologies) with random hexamers on the same RNA aliquots as for miRNA RT-qPCR. qPCR was performed using Power SYBR Green PCR Master Mix (Applied Biosystems, Life Technologies) and gene specific primers (IDT) in a final concentration of 0.3µM. Each qPCR reaction was performed in technical triplicates for each gene in each sample. miR-204 or miR-618 expression was normalized to RNU24 and miR-16 endogenous expression. EGFP expression was normalized to expression of TBP, SDHA and HMBS.

Sequences of primers used for qPCR:

| **Primer** | **Sequence (5'-3')** |
| --- | --- |
| EGFP_F | GTGGTGCCCATCCTGGTC |
| EGFP_R | CCGTCGTCCTTGAAGAAGAT |
| HMBS_F | GGCAATGCGGCTGCAA |
| HMBS_R | GGGTACCCACGCGAATCAC |
| SDHA_F | TGGGAACAAGAGGGCATCTG |
| SDHA_R | CCACCACTGCATCAAATTCATG |
| TBP_F | TGCACAGGAGCCAAGAGTGAA |
| TBP_R | CACATCACAGCTCCCCACCA |
